# A Hybrid Diffusion Model Enhances Multiparametric 3D Photoacoustic Computed Tomography

**DOI:** 10.1002/advs.202513624

**Published:** 2025-10-23

**Authors:** Hyunsu Jeong, Seunghun Oh, Seongwook Choi, Jiwoong Kim, Jinge Yang, Chulhong Kim

**Affiliations:** ^1^ Graduate School of Artificial Intelligence Departments of Electrical Engineering Convergence IT Engineering Mechanical Engineering Medical Science and Engineering and Medical Device Innovation Center Pohang University of Science and Technology (POSTECH) Pohang 37673 Republic of Korea; ^2^ Department of Radiology Molecular Imaging Program at Stanford (MIPS) School of Medicine Stanford University California 94034 USA; ^3^ Departments of Medical Engineering California Institute of Technology California 91125 USA

**Keywords:** deep learning, diffusion model, multiparametric imaging, photoacoustic computed tomography, photoacoustic imaging

## Abstract

Photoacoustic computed tomography (PACT) reveals biological structures, pharmacokinetics, and physiological functions. Although a premium PACT system with many ultrasound (US) transducers delivers high‐quality volumetric imaging, it suffers from high system costs and slow temporal resolution. Here, using a limited number of US elements, a hybrid diffusion model (HD‐PACT) is demonstrated that enhances dynamic multiparametric (structural, functional, and contrast‐enhanced) 3D PACT. Using just 256 out of the 1024 elements in a premier hemispherical US array for PACT, HD‐PACT improves structural images acquired in different planes, organisms, and wavelengths. In functional imaging, HD‐PACT enables 256‐element PACT to observe hypoxia, pharmacokinetics, and angiogenesis during tumor progression. Lastly, HD‐PACT is transferable to low‐end PACT (only 128 US elements), where it dynamically captures contrast‐free/enhanced organs, oxygen‐perturbed brains, and cardiac dynamics with high spatiotemporal resolution in live animals. It is believed that HD‐PACT will be valuable in oncology, cardiology, pharmacology, and endocrinology.

## Graphical abstract

The hybrid diffusion PACT (HD‐PACT) system enhances dynamic multiparametric information using only 128–256 ultrasound elements. By overcoming limited‐view artifacts, HD‐PACT refines structural and functional information such as oxygen saturation observed in 3D premium PACT with > 1000 elements. Through cost‐effective and faster multiparametric imaging, the system tracks tumor‐progression and captures dynamic contrast‐enhanced perfusion for oncology, cardiology, and pharmacology.

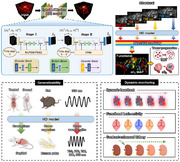

## 1 Introduction

Photoacoustic computed tomography (PACT) has attracted considerable attention as a non‐invasive biomedical imaging tool that provides multiparametric information—structural, molecular, and functional—for preclinical and clinical research.^[^ ^1^, ^2^, ^3^, ^4^, ^5^, ^6^, ^7^, ^8^, ^9^ ^]^ The photoacoustic (PA) effect describes the conversion of pulsed light energy into ultrasonic acoustic waves through repeated thermoelastic expansion^[^ ^10^, ^11^, ^12^, ^13^ ^]^ of the imaged target. The acoustic waves propagate to a detector and are then converted into PACT images.^[^ ^14^ ^]^ PACT has successfully obtained in vivo 3D structural images using endogenous contrasts (e.g., hemoglobin, lipid, and water) and also provided molecular information using exogenous contrasts, such as near‐infrared dye or gold nanoparticles.^[^ ^5^, ^15^, ^16^, ^17^, ^18^ ^]^ Further it also furnishes functional information by quantifying oxyhemoglobin (HbO), deoxyhemoglobin (HbR), and oxygen saturation (sO_2_) with multi‐wavelength illumination.^[^ ^15^, ^19^, ^20^, ^21^, ^22^, ^23^, ^24^ ^]^ Thus, PACT has been widely employed for preclinical and clinical applications in endocrinology,^[^ ^25^, ^26^ ^]^ oncology,^[^ ^27^, ^28^, ^29^, ^30^ ^]^ pharmacology,^[^ ^31^, ^32^, ^33^, ^34^ ^]^ and cardiology.^[^ ^35^, ^36^, ^37^ ^]^

Since PA waves are emitted omnidirectionally from a target, capturing these signals in a spherical configuration minimizes limited‐view artifacts and ensures high‐quality imaging outcomes.^[^ ^38^ ^]^ Therefore, premium PACT systems have predominantly adopted a hemispherical detection design^[^ ^39^, ^40^, ^41^, ^42^, ^43^, ^44^ ^]^ with a hemispherical ultrasound (US) transducer array and a multi‐channel data acquisition (DAQ) system to accomplish real‐time volumetric PACT and multiparametric dynamic imaging. Such a hemispherical US transducer array, characterized by a large aperture and numerous elements, demonstrably improves image quality. Nonetheless, rapid imaging demands a relatively high number of DAQ channels, which inevitably increases the system cost. Although multiplexing PA signals can decrease the required number of DAQ channels and reduce costs, it inevitably sacrifices imaging speed.^[^ ^45^ ^]^

Here, we introduce a diffusion‐based deep learning (DL) model, called hybrid diffusion PACT (HD‐PACT), which provides dynamic multiparametric (structural, functional, and contrast‐enhanced) volumetric information with a limited number of US elements (as few as 128 out of the 1024 elements in a premier hemispherical US array for PACT). HD‐PACT is a model‐augmented PACT system that integrates standard PACT hardware and a hybrid diffusion (HD) model to recover full‐array images from arrays with fewer‐elements. The “hybrid” attribute comes from our proposed module, called efficient‐hybrid mamba, which combines Mamba^[^ ^46^ ^]^ and an efficient self‐attention^[^ ^47^ ^]^ HD‐PACT makes three key contributions. First, HD‐PACT captures high‐resolution structural 3D PACT images in live animals and humans using a sparsely selected 256 elements in the hemispherical array (referred to as sparse sampling). The enhanced HD‐PACT image quality is comparable to that from a complete set of 1024 US elements (full sampling). HD‐PACT is validated across multiple imaging planes and organisms, and by using optical wavelengths that were unseen during training stages, thus demonstrating its potential to take advantage of reduced data throughput in various PA imaging fields.^[^ ^48^, ^49^ ^]^ Second, HD‐PACT provides quantitative structural and functional information (e.g., sO_2_ and total hemoglobin concentration (HbT)), demonstrated by monitoring angiogenesis and hypoxia in a tumor‐bearing region during cancer progression. HD‐PACT preserves and enhances per‐wavelength images, enabling spectral unmixing of multi‐wavelength datasets. The resulting functional information is computed from the restored/unmixed images, thereby serving as applications of HD‐PACT. Lastly, transfer learning allows dynamic multiparametric imaging by capturing structural, functional, and contrast‐enhanced information (e.g., neurological functional activities, physiological heart signals, and contrast‐free/enhanced organs) using a very limited number of elements (128 elements). In summary, HD‐PACT can overcome the limitations of low‐specification PA hardware, enabling valuable contributions to cancer monitoring, endocrinology, oncology, pharmacology, and cardiology.

## 2 Results

### 2.1 Hybrid Diffusion Model Enhanced PACT (HD‐PACT)

The PACT data used in this work were obtained with a previously developed PACT system (Figure S1a, Supporting Information).^[^ ^50^, ^51^, ^52^ ^]^ The PACT system employed a 1024‐element hemispherical transducer array with a 2.02 MHz center frequency, a 20 Hz optical parametric oscillator laser, and a 256‐channel data acquisition (DAQ) system with a 4:1 multiplexing board. At a fixed transducer position, PACT obtains a single volumetric image covering a field‐of‐view (FOV) of 12.8 mm × 12.8 mm × 12.8 mm. Large FOV PACT imaging was performed by stitching together single volumes acquired through raster scanning (Figure S1b, Supporting Information). Due to the small number of DAQ channels, we collected high‐definition PACT data with a frame rate 5 Hz by taking the PA signals from all 1024 elements of the transducer array (referred to as “1024‐full” data). To reduce data throughput, the PACT system employed techniques that sample reduced numbers of detectors, including sparse or cluster sampling (detailed in the Experimental Section). PACT single‐volume in vivo images of a rat corresponding to each sampling method show their characteristic image qualities (Figure S2a, Supporting Information). Sparse sampling preserves the detection aperture but increases detection sparsity, causing radial‐streak artifacts. In contrast, cluster sampling avoids sparsity but causes blurred images due to the small detection aperture (Figure S2b, Supporting Information). However, cluster sampling can advantageously capture data from the entire element subset simultaneously because of the US‐element arrangement, thus accelerating imaging speed.^[^ ^50^ ^]^

**Figure 1:**
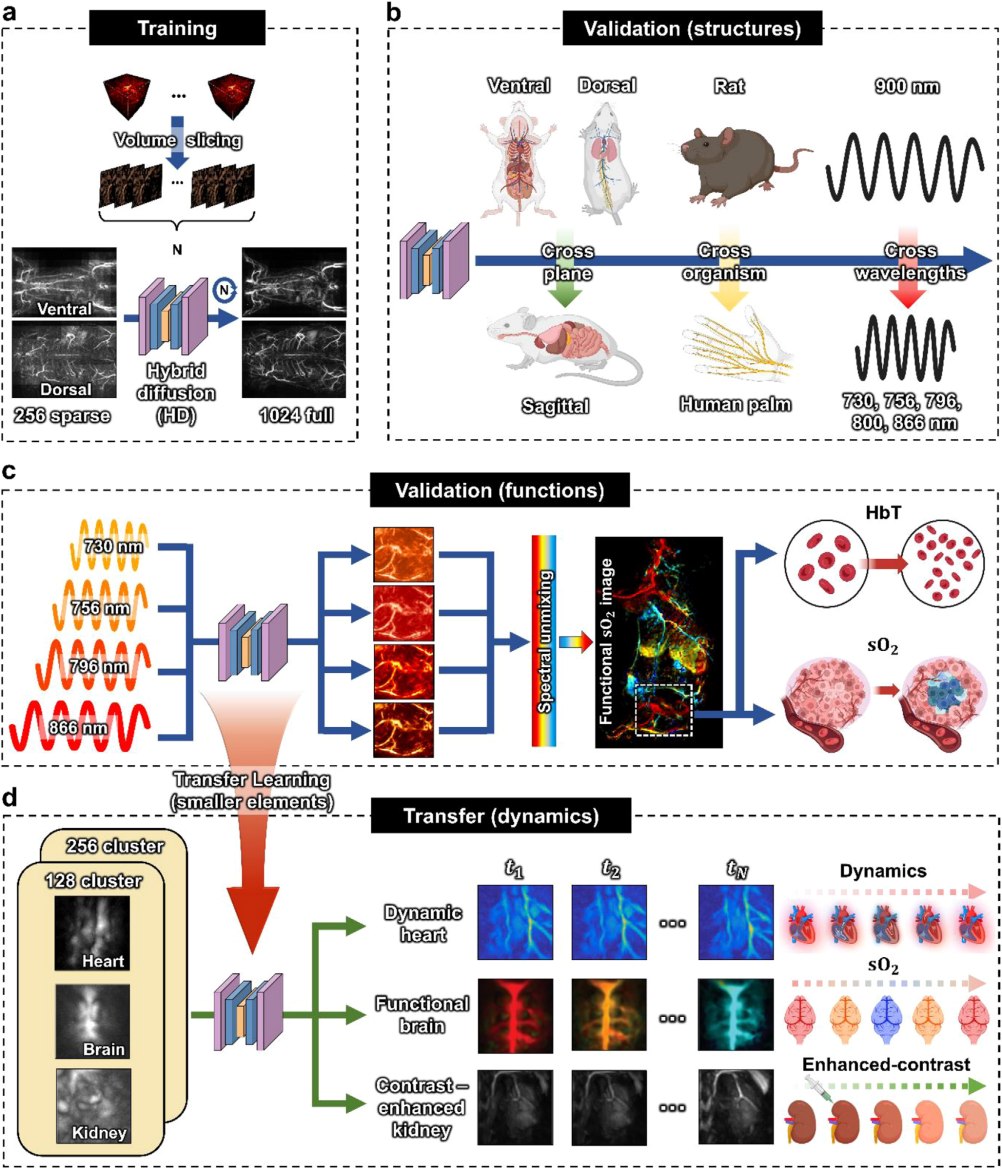
Overall schematic overview of HD‐PACT. a) Training stage for sparsely activated 256 ultrasound (US) elements (256‐sparse elements) out of 1024 full elements. We acquire volumetric data from both the 1024‐full and 256‐sparse element configurations. Volume slicing provides three adjacent slices as input to the hybrid diffusion (HD) model. The training dataset consists of photoacoustic computed tomography images acquired from the ventral and dorsal planes of rats at a 900 nm wavelength. b) Structural validation of the HD network. For generalization, the HD model is validated on a different plane (i.e., the sagittal plane), another organism (a human palm), and at different wavelengths (730, 756, 796, 800, and 866 nm). c) Functional validation of the HD model on a tumor‐bearing mouse. Four unseen wavelengths (730, 756, 796, and 866 nm) from the training data provide time‐dependent functional information through spectral unmixing, capturing sO_2_ and HbT during tumor progression. d) Transfer learning for cluster sampling. The transfer HD model offers dynamic multiparametric information (e.g., structural, functional, and contrast‐enhanced) through physiological signals of the heart, oxygen‐sensitive brain, and contrast‐free/enhanced organs of the imaged animals. Specifically, the transfer HD model enables high‐speed visualization of cardiac cycles, including the systolic and diastolic phases, it detects functional brain activity via sO_2_ dynamics, and it restores vascular and organ structures both with and without contrast agents, all using only 128 elements, 1/8 of the full number of US elements. The schematic illustrations (e.g., rat, mouse, human palm, and wavelength) and biological diagrams (e.g., cellular and anatomical structures) were created with BioRender.com.

As shown in the overall schematic in **Figure** **1**, we trained HD‐PACT using 256‐sparse and 1024‐full PACT paired images. The model utilizes three adjacent slices in a volumetric data set —the previous, current, and next slices—as the input data for training (Figure 1a). PA‐optimized noise is injected during the forward process, and our model is trained to remove it in the reverse process. The training process of HD‐PACT is described in greater detail in the next section. To generalize its performance, HD‐PACT was validated across different plane, organism, and wavelengths (Figure 1b). Although HD‐PACT was trained using only 900‐nm PA images of rats in the ventral and dorsal planes, the model also enhances structural information in another plane (the sagittal plane), another organism (a human palm), and at other wavelengths (730, 756, 796, 800, and 866 nm) (Table S1, Supporting Information). This generalizability allows the network to accurately reconstruct PACT images at multiple wavelengths, which is essential for spectral unmixing. As a result, our network effectively enables one of the core capabilities of PACT, functional imaging, by supporting robust spectral unmixing. Consequently, HD‐PACT to extract functional information (HbO, HbR, and sO_2_) by spectrally unmixing the four‐wavelength datasets (taken at 730, 756, 796, and 866 nm). HD‐PACT can monitor tumor angiogenesis and hypoxia during tumor progression by observing HbT and sO_2_ (Figure 1c). Finally, transfer learning with the 256‐sparse pretrained model allows HD‐PACT to improve the cluster‐sampled data, capturing dynamic multiparametric information about the heart, brain, and organs (Figure 1d).

### 2.2 Hybrid Diffusion (HD) Model

**Figure 2:**
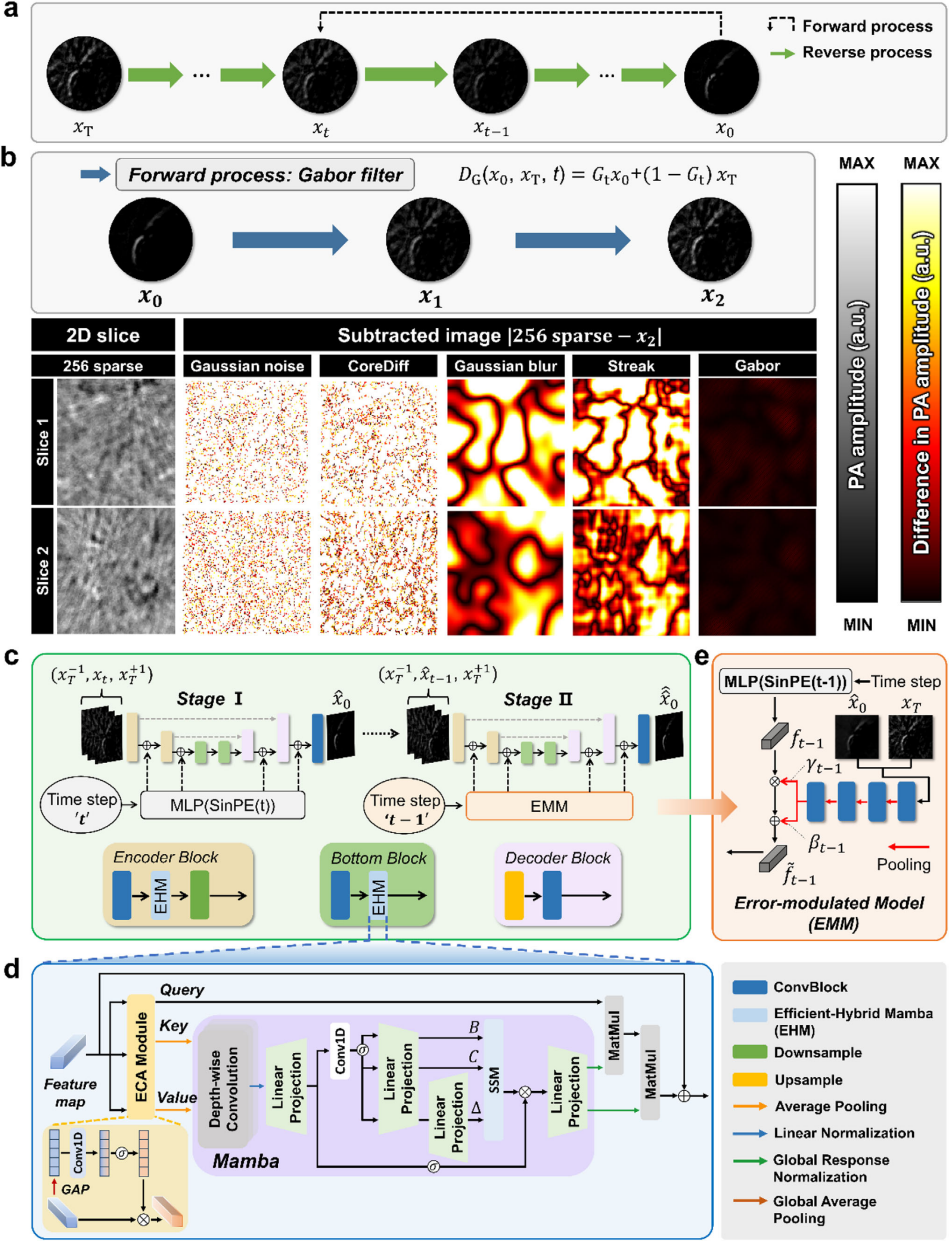
Hybrid diffusion (HD) model. a) Forward and reverse processes. In the forward process, the HD model injects a specific type of noise repeatedly over *T* iterations. During training, the HD model performs a reverse process that predicts the injected noise and removes it over *T* iterations, thereby restoring the original image. The HD model takes a noisy image *x_T_* as input and performs *T* iterative steps to eliminate the noise, resulting in a denoised image *x*_0_. b) Difference maps between a 256‐sparse image and various types of degraded images during the forward process. c) The HD model uses three adjacent slices as an input and estimates a denoised image in a two‐stage process. In stage I, time‐embedding features for the *t*‐th denoising step are reflected using a multi‐layer perceptron and sinusoidal position embedding (SinPE(*t*)); in stage II, feature information from the (*t*–1)‐th step is applied via the Error‐Modulated Model (EMM). d) The efficient‐hybrid mamba (EHM) module consists of efficient channel attention (ECA),^[^ ^54^ ^]^ Mamba with a state space model (SSM),^[^ ^46^ ^]^ and efficient self‐attention (ESA).^[^ ^47^ ^]^ ECA and Mamba extract channel‐attentive and selectively important information. ESA applies self‐attention using reduced key and value feature maps to consider long‐range dependency. e) EMM for correcting the misalignment between the *t*–1 time‐embedding vector and the input ${\hat{x}}_{t-1}$ at stage II using a shallow network. The EMM employs the errors between the previous result ${\hat{x}}_{0}$ and *x_T_*, modulating the time‐step *t*–1 embedding for the stage II.

Figure 2 illustrates the overall training procedure and architecture of the HD model. As the HD model builds upon the diffusion‐based CoreDiff^[^ ^53^ ^]^ framework, its training involves both a forward and reverse process during training, while only the reverse process is executed during inference (**Figure** **2**a). We used a PA‐optimized forward process to replace the CoreDiff^[^ ^53^ ^]^ forward designed for X‐ray computed tomography. In our study, the Gabor filter effectively simulates noise patterns observed in 256‐sparse images. These noises arise from distortions along the time‐of‐flight paths of acoustic waves.^[^ ^55^ ^]^ The Gabor filter‐based forward process selectively removes specific frequency components, approximating the degradation of texture observed in the 256‐sparse image. As shown in Figure 2b and Figure S3 (Supporting Information), our forward method most accurately simulates sparse‐element noise and yields more suitable degradation than Gaussian noise, Gaussian blur, streak filter‐based forward, and CoreDiff^[^ ^53^ ^]^ forward in the PA domain. Unlike streak filter focusing on linear patterns, the Gabor filter exhibits directional and frequency‐based selectivity during the forward process, thereby preserving the original structure throughout the reverse process (Figure S3, Supporting Information). Furthermore, the Gabor filter enables model inference with only two time steps without sacrificing performance. Compared to the 1000‐step, our two‐step model achieves a peak signal‐to‐noise ratio (PSNR) of a 42.20±5.19 dB (a 0.44 dB improvement) and a structural similarity index map (SSIM) of 0.9630 ± 0.0281 (a 0.0006 improvement). Most notably, the two‐step model reduces the inference time from 11.451 to 0.026 s (Figure S4, Supporting Information).

In the reverse process, the HD model denoises the artifacts in sparsely sampled PACT images by progressively predicting and removing the noise injected at time *T* during the forward step (Figure 2a). The efficient‐hybrid mamba (EHM) module is embedded in the encoder and bottleneck block (Figure 2c). The EHM goes through three steps: efficient channel attention (ECA),^[^ ^54^ ^]^ efficient self‐attention (ESA), and Mamba. First, the feature maps pass through the ECA module with 1 × 1 × 1 convolution to efficiently learn the relative importance of each channel. Second, the key and value feature maps are reduced to apply the ESA mechanism. Although self‐attention extracts global features considering long‐range dependency, it suffers from high computational cost and poor inductive bias, requiring increased training time and large datasets. Since self‐attention has a low‐rank property,^[^ ^47^ ^]^ key and value maps are reduced to efficiently apply self‐attention computation, achieving computational efficiency without mitigating performance on 256‐sparse data (Figure S4, Supporting Information). Third, the reduced maps are processed by the Mamba module to selectively extract more essential information from long sequences. Subsequently, the ESA operation that applies self‐attention using reduced key and value maps generates the feature map by considering the long‐range dependency of the calibrated key and value maps (Figure 2d). The key components, such as ECA, ESA, and Mamba improve performance by properly removing the noise in the 256‐sparse PA image (Table S2, Supporting Information). Furthermore, the Error‐Modulated Model (EMM) is used to adjust the misalignment of time‐embedding at Stage II (Figure 2e). By incorporating adjacent slices as input data, the HD model leverages consistent structural patterns to extract structural information more effectively, overcoming the limitations of training on a single noisy 2D slice (Figure 2c). Detailed explanations of the network and training process are given in the Methods section.

### 2.3 HD Enhanced Structural PACT Information from 256‐Sparse Elements

**Figure 3:**
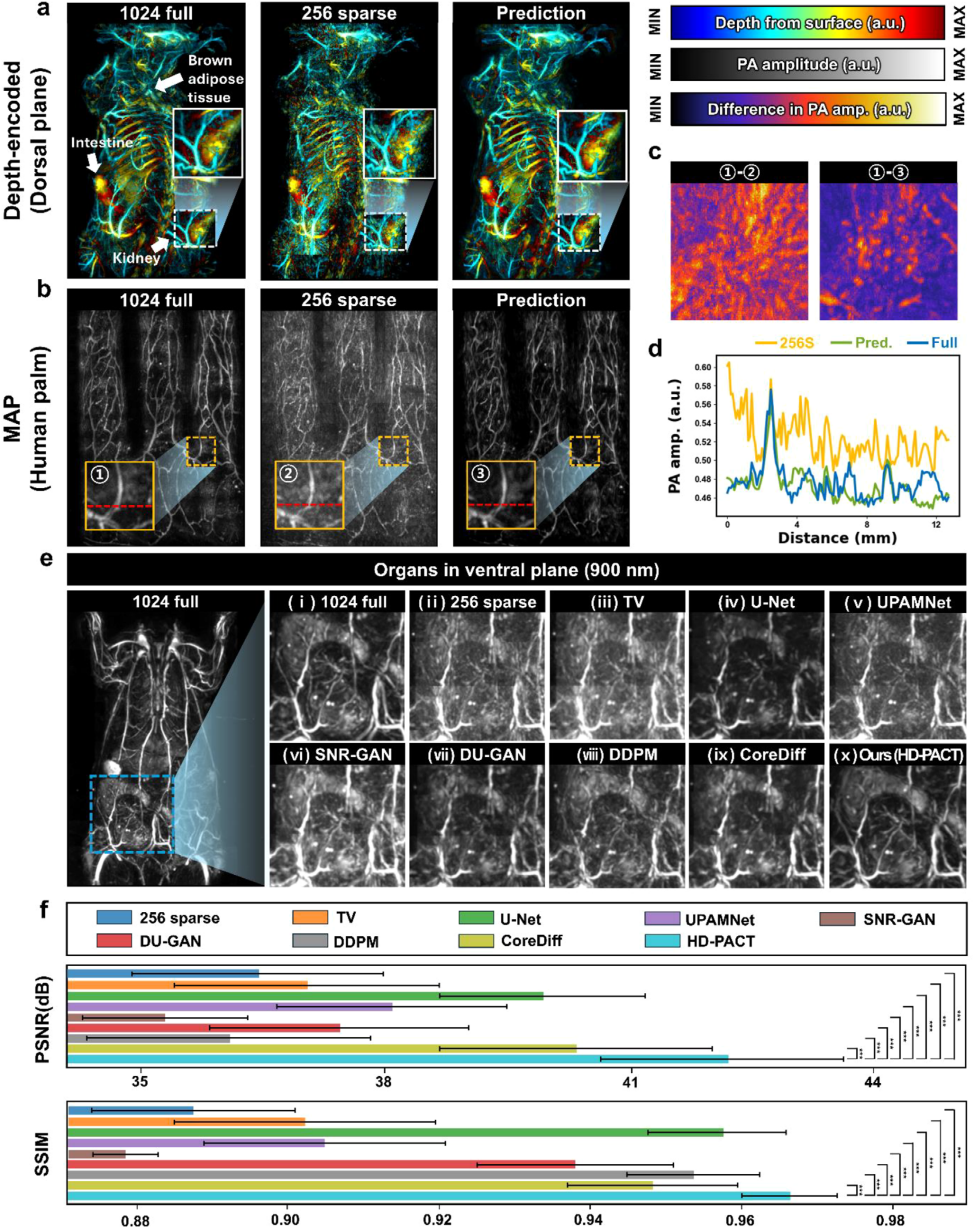
Structural enhancement from 256‐sparse HD‐PACT images.a) Depth‐encoded whole‐body dorsal plane PACT images reconstructed with 1024‐full and 256‐sparse data, and the corresponding HD predicted image. The region of interest (a kidney) is shown enlarged in the white dashed boxes. b) Maximum amplitude projection (MAP) images from 1024‐full and 256‐sparse data from a human palm, and the HD prediction. The region of interest (a blood vessel) is bounded by the dashed yellow square and shown zoomed out in the yellow‐bordered boxes. c) Two difference maps for the yellow‐zoomed area, in which the 256‐sparse image and the prediction, respectively, are subtracted from the 1024‐full image. d) Comparison of red‐line profiles for the 1024‐full, 256‐sparse, and prediction images. e) Qualitatively comparable images from 1024‐full (ground truth, GT), 256‐sparse (input), TV,^[^ ^56^ ^]^ U‐Net,^[^ ^57^ ^]^ UPAMNet,^[^ ^58^ ^]^ SNR‐GAN,^[^ ^59^ ^]^ DU‐GAN,^[^ ^60^ ^]^ DDPM,^[^ ^61^ ^]^ CoreDiff,^[^ ^53^ ^]^ and our HD‐PACT. The organs are extracted from a whole‐body rat view in a ventral plane. f) Quantitative comparisons of PSNR and SSIM calculated for the predicted results (256‐sparse, TV, U‐Net, UPAMNet, SNR‐GAN, DU‐GAN, DDPM, CoreDiff, and HD‐PACT) versus their corresponding 1024‐full (GT). For all results, a paired *t*‐test shows a statistical difference between HD‐PACT and the other models (*p* < 0.001).

**Figure** **3** shows that HD‐PACT improves the structural image quality of 256‐sparse PACT data. Figure 3a presents depth‐encoded whole‐body PACT images captured with 1024‐full and 256‐sparse elements, and the corresponding HD‐PACT predicted image of a live rat at 900 nm in the dorsal plane. The 1024‐full image, serving as the ground truth (GT), exhibits superior spatial resolution and structural details. In contrast, the 256‐sparse image is notably degraded, with structural distortions and loss of fine vascular details. The HD‐PACT prediction effectively restores the 256‐sparse image quality to the level of the 1024‐full image, clearly revealing anatomical features such as the intestine, brown adipose tissue, and the kidneys. Notably, the kidney, shown in white‐dashed boxes, displays reduced radial‐streaky artifacts in the 256‐sparse image. HD‐PACT also recovers ventral anatomical structures, including the sternum, liver, spleen, cecum, intestine, and a blood vessel (Figure S5, Supporting Information).

Since DL‐based models are sensitive to the heterogeneity between training and test data, we used different planes, organisms, and optical wavelengths to validate HD‐PACT. Compared to the ventral and dorsal planes used in training, the 730 nm sagittal MAP images exhibited different PA intensities and organ shapes. Nevertheless, HD‐PACT improves the MAP image quality even in the different plane and wavelength images (Figure S6, Supporting Information). HD‐PACT was further validated across different organisms by using in vivo human palm data. As shown in Figure 3b, the 256‐sparse image contains noise that obscures the blood vessels, while the HD prediction closely resembles the 1024‐full image, recovering vascular structure and reducing noise artifacts. Since HD‐PACT learns to remove the radial‐streak noise injected by the Gabor‐matched forward with three adjacent slices, the approach reduces the background noise more than the 1024‐full image. These capabilities indicate its ability to restore missing details from sparse acquisitions. The difference map of yellow‐dashed boxes manifests that the HD‐PACT dramatically reduces detailed noise in the palm vessels (Figure 3c). The performance of HD‐PACT was further validated by analyzing the line profiles traced along the dashed red lines (Figure 3d).

We compared HD‐PACT's performance with four types of denoising methods: 1) an iterative denoising algorithm, Total Variation (TV);^[^ ^56^ ^]^ 2) two CNN‐based methods, U‐Net^[^ ^57^ ^]^ and UPAMNet;^[^ ^58^ ^]^ 3) two GAN‐based methods, SNR‐GAN^[^ ^59^ ^]^ and DU‐GAN;^60^and 4) two diffusion‐based method, DDPM^[^ ^61^ ^]^ and CoreDiff.^[^ ^53^ ^]^ HD‐PACT demonstrated the state‐of‐the‐art performance, most clearly representing the kidney and vascular details (Figure 3e; Figure S7, Supporting Information). Quantitative metrics with paired *t*‐tests showed HD‐PACT outperformed 256‐sparse data by 5.77 dB in PSNR and 0.0755 in SSIM (Figure 3f; Table S3, Supporting Information). The TV method shows the worst performance under high‐noise conditions. Although U‐Net achieves a high SSIM by denoising speckle noise, the PSNR remains relatively low due to slight blurring and intensity mismatches at the pixel level. Compared to HD‐PACT, all the GAN‐based methods exhibit consistently lower performance in both PSNR and SSIM. Our baseline model, CoreDiff,^[^ ^53^ ^]^ achieves competitive performance (PSNR, 40.36 ± 5.54 dB; SSIM, 0.9491 ± 0.0413), yet still clearly lagged HD‐PACT in both metrics, demonstrating the superior performance and robustness of our approach. Furthermore, HD‐PACT also outperforms other methods in individual cases, such as sagittal whole‐body mouse imaging and human palm imaging (Table S4, Supporting Information).

### 2.4 HD Enhanced Multiparametric PACT Information for Tumor Monitoring

**Figure 4:**
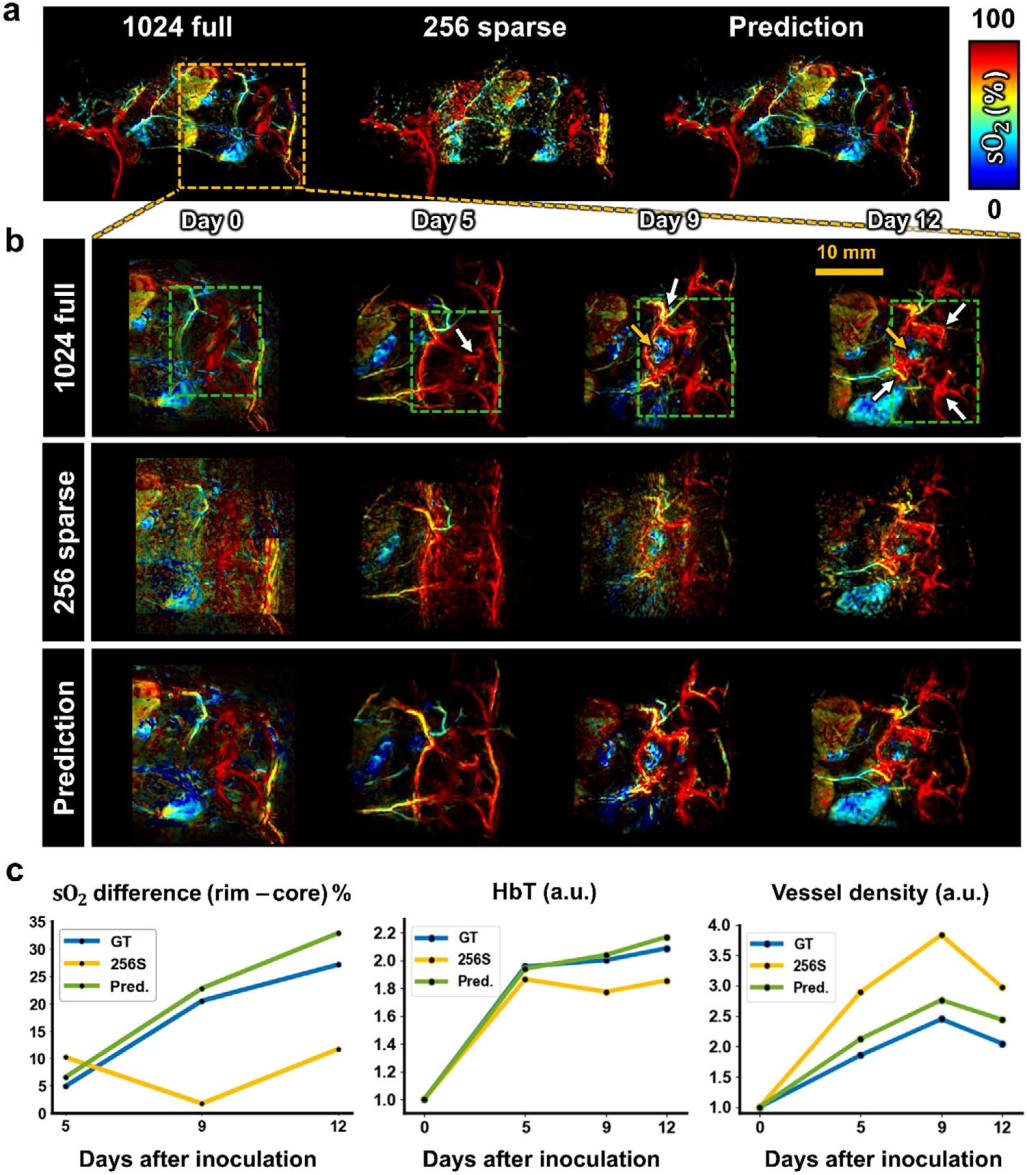
HD enhanced multiparametric PACT imaging of tumor‐bearing mice. a) Whole‐body PA sO_2_ images of a mouse captured with 1024 and 256 elements immediately after 4T1 breast cancer cell inoculation (Day 0), and the HD predicted image. The yellow‐dashed box indicates the region of interest (ROI) containing the tumor and is magnified in Figure 4b. b) Time‐lapse PA sO_2_ images of the tumor ROI from 1024‐full elements and 256‐sparse elements, with corresponding HD predictions on days 0, 5, 9, and 12 after inoculation. The ROI is bounded by green‐dashed lines. White arrows show regions of tumor‐induced angiogenesis, and yellow arrows on days 9 and 12 indicate the hypoxic region in the tumor core. c) Quantitative analysis of multiparametric tumor progression information: The increasing difference in sO_2_ between the tumor rim and core indicates oxygen heterogeneity, and the changes in HbT and vessel density over time show the trends in blood supply to the tumor region, providing structural information relevant to angiogenic activity. Comparisons are shown among GT (1024‐full data), 256S (256‐sparse data), and Pred (HD prediction).

PACT excels in providing functional information through spectral unmixing of multi‐wavelength PA images. We applied HD‐PACT to analyze PA multiparametric biomarkers in tumor‐bearing mouse inoculated with 4T1 breast cancer cells. As seen in **Figure** **4**a,b, at day 0, 1024‐full HD‐PACT provided a high‐resolution PA sO_2_ image of the whole mouse body, and on days 5, 9, and 12 after inoculation. The time‐lapse functional images obtained on days 5, 9, and 12 demonstrate tumor‐induced angiogenesis, indicated by high sO_2_ levels in the newly formed vessels (white arrows in Figure 4b). The tumor core, indicated by yellow arrows in Figure 4b, presents low sO_2_ levels on days 9 and 12, suggesting that while early angiogenesis raises peripheral sO_2_, tumor growth eventually induces central hypoxia during tumor progression. These physiological observations agree well with previously reported results.^[^ ^51^ ^]^ On the other hand, the 256‐sparse PACT shows significant degradation in functional images, exhibiting blurred boundaries, fragmented vascular networks, and missing fine vessel structures. These degraded images were improved by the HD model, even though PA images of a tumor‐bearing mouse at four wavelengths (730, 756, 796, and 866 nm) were not included in the training. As shown in Figure 4b, the HD model successfully suppresses the artifacts and restores the sO_2_ values, clearly capturing subtle changes in vessel oxygenation after the inoculation. Furthermore, while it is challenging to distinguish tumor‐core hypoxia (shown as the white‐dashed circle) in the cross‐section of a 256‐sparse image, the HD prediction clearly reveals the sO_2_‐difference boundary between the core and rim, similar to the 1024‐full image (Figure S8, Supporting Information).

We further quantified the functional parameters during the tumor progression (Figure 4c). Although the sO_2_ difference shows significant discrepancy between the 256‐sparse and 1024‐full images on days 9 and 12, our model reduces these deviations and follows the inclination of the 1024‐full sO_2_ difference. In addition to the sO_2_ difference, the HD model also performs well in qualifying HbT and vessel density as additional biomarkers for tumor progression. During the tumor growth, the HbT values in both 1024‐full data and the HD prediction increase continuously, approximately doubling from day 0 ​​to day 12. However, the 256‐sparse acquisition introduces noise, causing inaccurate HbT quantification that deviates from the 1024‐full data trend. For the vessel density, the HD prediction shows an ≈25% increase on day 9. In contrast, a 40% increase is observed in the 256‐sparse MAP image, primarily due to high‐intensity noise resembling blood vessels, leading to overestimation of the vessel density. Overall, these findings reveal that our model can monitor angiogenesis and hypoxia with PACT systems using 256‐sparse elements, reconstructing images that closely align with observations from 1024‐full data.

### 2.5 HD Enhanced Dynamic PACT Imaging Via Transferability

Although 1024‐element PACT provides high‐resolution images, its hardware complexity (e.g., 1024 US elements vs 256 DAQ channels) limits fast dynamic monitoring. Using lower‐specification PACT with fewer elements accommodates cost‐effective hardware (e.g., low‐channel DAQ and fewer US elements) and accelerates imaging speed, but suffers from low spatial resolution. Here, HD‐PACT provided enhanced multiparametric dynamic information from a cluster of only 128 elements. To improve the quality of the 128‐cluster noisy data, we applied transfer learning using a pretrained model on 256‐sparse data, reducing both the data and training requirements. Compared to the original 256‐sparse setup, the transfer learning used only 1/9 of the training dataset and only 1/11 as many training iterations, demonstrating efficient adaptation to constrained data and computational resources.

**Figure 5:**
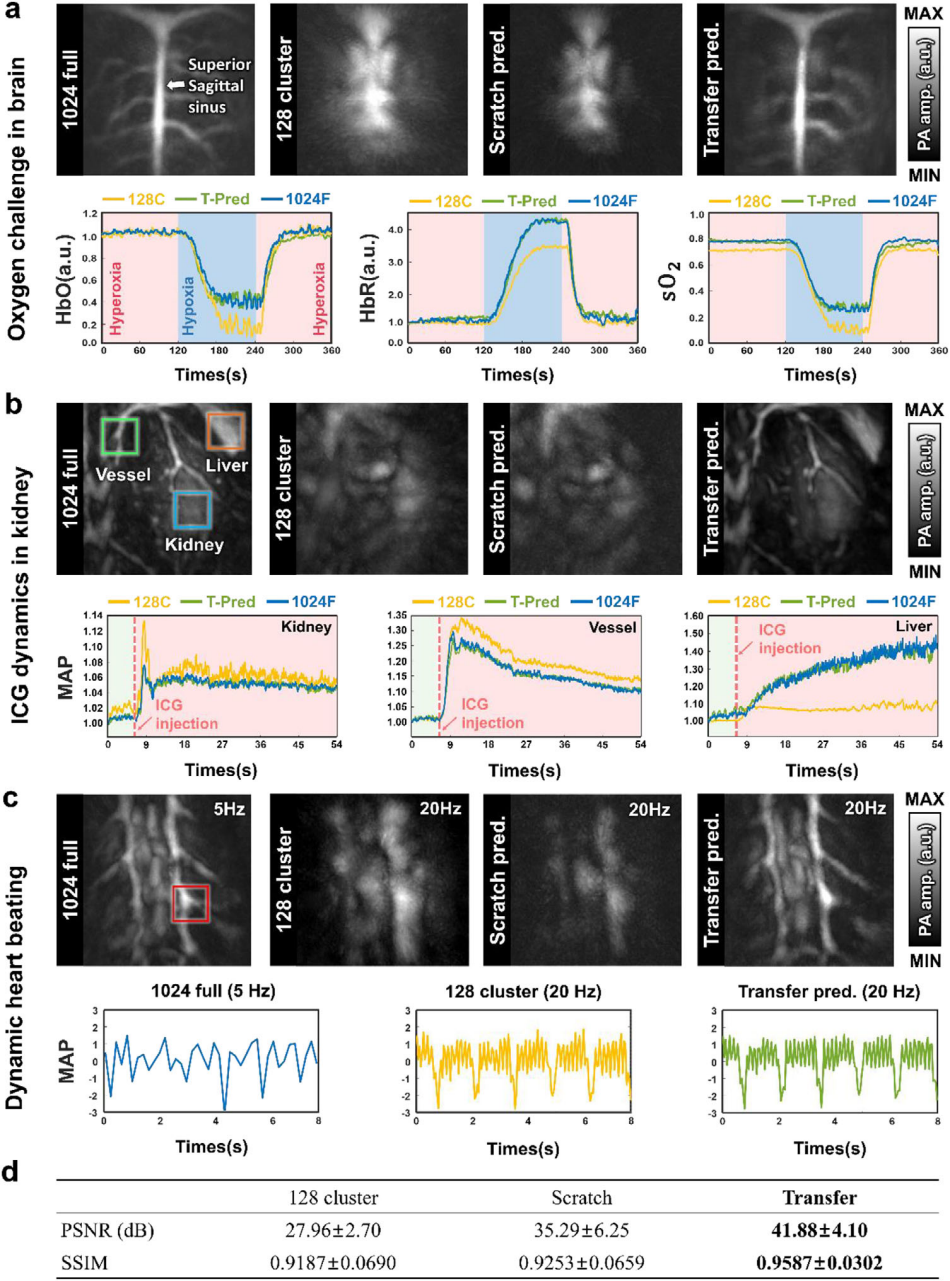
Dynamic PACT imaging through transfer learning from 128‐cluster to 1024‐full datasets. a) MAP images of a rat's brain from 1024‐full elements (1024F) and 128‐cluster elements (128C), with the prediction of the scratch‐trained HD model without transfer learning and the prediction of the transfer HD model (T‐pred). Time‐lapse changes in HbO, HbR, and sO_2_ signals during an oxygen challenge are also shown. b) MAP images of the kidney (blue box), blood vessel (green box), and liver (orange box), with corresponding time‐lapse PA signal changes following ICG injection. c) MAP images of rat's heart reconstructed from 1024‐full and 128‐cluster data, with time‐lapse PA signals from the heart (red box). Note that due to the limitations of the hardware system, the maximum achievable frame rate for images with 1024‐full elements is 5 Hz, while the prediction of the transfer HD from the 128‐cluster data is 20 Hz. d) Quantitative comparison of the 128‐cluster data, the prediction of the scratch HD model, and the prediction of the transfer HD model to the 1024‐full data.

**Figure** **5**a presents cortical vessel PACT MAP images and the time‐lapse functional signals (HbO, HbR, and sO_2_) of rat's brain during an oxygen challenge. The oxygen challenge involved alternating the inhaled gas between 90% oxygen/10% nitrogen (hyperoxia) and 10% oxygen/90% nitrogen (hypoxia) to induce sO_2_ changes. The 1024‐full image shows clear cerebrovascular vessels extending from the superior sagittal sinus while the 128‐cluster image appears to show blurry vessels. The scratch HD model (trained from scratch without transfer learning) fails to reconstruct the cerebrovascular vessels, indicating limited data is insufficient for effective cortical vascular reconstruction. In contrast, the transfer HD model restores vessel clarity and captures dynamic functional parameters (HbO, HbR, and sO_2_). It also reflects trends consistent with the 1024‐full data during an oxygen challenge under both hyperoxia and hypoxia. The entire functional neuroimaging process is visualized in Video S1 (Supporting Information).

Figure 5b displays clear 1024‐full MAP images of a rat's kidney, blood vessel, and liver. However, the accompanying 128‐cluster image and the scratch HD prediction both exhibit severe blurring, indicating that training from scratch offers no meaningful improvement. The transfer HD model, however, significantly enhances image quality, delineating organ boundaries and restoring structural clarity. It also captures contrast‐enhanced dynamics by tracking the kinetics of injected indocyanine green (ICG), closely matching the 1024‐full data. Early after ICG injection, the 1024‐full PA signals increase in the vessel and kidney, with a subsequent rise in the liver. The transfer HD model reproduces these dynamic patterns while preserving finer contrast details. The perfusion of ICG in the kidney is further evident in Video S2 (Supporting Information).

We also confirmed that the HD model enables faster high‐resolution cardiac imaging with fewer transducer elements (Figure 5c). While using 1024‐full elements provides superior image quality, its DAQ channel limits the imaging speed to 5 Hz, restricting the capture of rapid in vivo physiological dynamics of cardiac function. In contrast, using 128‐cluster elements allows 20 Hz imaging in the current configuration. As shown in Figure 5c and Figure S9 (Supporting Information), the transfer HD model enhances structural detail at 20 Hz and dynamically captures cardiac physiological patterns. Video S3 (Supporting Information) further visualizes these dynamics. While the 256‐channel DAQ increased the imaging speed by four times, the 128‐channel DAQ allows 128‐cluster PACT to theoretically operate eight times faster than 1024‐full PACT. Therefore, our results suggest that the transfer HD model can feasibly support practical applications with a more cost‐effective system.

Quantitatively, the transfer HD model achieved superior performance to the 128‐cluster and the scratch HD model (*p* < 0.001) (Figure 5d). Table S5 (Supporting Information) provides organ‐wise results. We obtained similar results on the 256‐cluster dataset. Figure S10 (Supporting Information) presents the dynamics results from 256‐cluster data on the brain, organs, and heart, obtained using the same protocols as those in the 128‐cluster experiments. Videos S4–S6 (Supporting Information) respectively display functional brain activity under oxygen challenge, contrast‐enhanced kidney perfusion, and high‐speed cardiac dynamics from 256‐cluster data. These results confirm that the transfer HD model consistently enhances image quality and functional accuracy, highlighting its robust transferability across element configurations.

## 3 Discussion

This paper introduces HD‐PACT, a diffusion‐based framework designed to overcome the hardware limitations of PACT. HD‐PACT provides high‐quality structural, functional, and dynamic imaging using significantly fewer ultrasound (US) elements—only 128 or 256 out of 1024—without compromising spatial and temporal resolution (Figure S11, Supporting Information). By using Gabor filter‐based degradation as a PA‐optimized forward process, we reduce the inference time, facilitating the integration of advanced computation modules such as Efficient‐Hybrid Mamba (EHM). In addition, Figure S12 (Supporting Information) shows why fewer steps with well‐defined forward perform better. Since the reverse process cannot perfectly remove the noise, step‐wise error is progressively accumulated. With a Gabor filter, when T = 1000, the per‐step increments |*x_n_* − *x*_*n* + 1_| (*n* ≥ 2) are negligible, leading to a long sequence of infinitesimal updates toward *x*_1_ and a large jump between *x*_1_ and *x*_0_ that degrades the final reconstruction. In contrast, with T = 2, the model learns a visibly‐noisy change from *x*_2_ to *x*_1_. This allows the model to predict the noise pattern between *x*_1_ and *x*_0_, supporting minimum‐forward steps without sacrificing performance. Further, 3D contextual information from three adjacent slices aids in differentiating noise from structure and effectively removing the noise. However, there is still a limitation on the real‐time processing of whole‐body images at the volume level, thereby demanding high‐specification GPU or lightweight DL modules for better throughput and computational efficiency.

We verified the structural and functional imaging capabilities of HD‐PACT by capturing 256‐sparse whole‐body rat images on ventral and dorsal planes at a 900 nm laser wavelength. HD‐PACT effectively removed noise while preserving depth information. This performance was also generalized to different planes, organisms, and wavelengths, including sagittal rat images at 730 nm and human palm images at 800 nm. The functional outputs for tracking angiogenesis and hypoxia during tumor progression closely resembled those from 1024‐full images. HD‐PACT also refined sO_2_ measurements by reducing radial‐streaky noise, enabling clear differentiation between the tumor core and rim. The improved sO_2_ difference, HbT, and vessel density information show the modality's potential for preclinical studies, even using low‐specification PACT. Furthermore, using the SNR and CNR for identical ROIs, we quantified not only the depth‐ and tissue‐dependent performance, but also the noise‐level effects in HD‐PACT. The SNR and CNR decreased with depth, and CNR differed by organ at matched depths while SNR was similar. Weaker signals in angiogenesis reduced the SNR and CNR, with prediction improving but not fully restoring CNR under extremely weak signals (Figures S13 and S14, Supporting Information). However, work is still needed to eliminate or compensate for the overestimation of the reduced sO_2_ in tumor regions. Future work should focus on incorporating physical and physiological constraints into the model training process to ensure biologically faithful predictions.

HD‐PACT can extract dynamical (structural, functional, and contrast‐enhanced) information using only 128 elements. Its transferability enables efficient use of pretrained features, reducing the need for extensive data and training.^[^ ^62^ ^]^ Validation on 128‐cluster PACT data shows improved imaging of anatomical structures, even with blurred vascular and organ boundaries. While 256‐sparse sampling introduces streak‐like noise but preserves vascular and organ edges, cluster sampling reduces noise but blurs structures by removing high‐spatial‐frequency content, which hinders scratch training. Pretraining on 256‐sparse data transfers structural priors (e.g., vessel edges and continuity) that enable effective artifact suppression on cluster sampling. The transfer model also captures molecular information, such as contrast‐enhanced dynamics, and functional brain responses under an oxygen challenge. These findings indicate that our HD model, pretrained on 256‐sparse data, successfully transferred to cluster‐sampled data with a reduced detecting aperture, still extracting comparable multiparametric information. Although HD‐PACT was tested on a 256‐channel MUX and with a 20‐Hz laser, the outstanding outcomes suggest the feasibility of using a MUX with fewer channels and a faster laser, enabling faster and cost‐effective imaging.

In conclusion, we have developed and demonstrated HD‐PACT, a diffusion‐based model that addresses hardware limitations in volumetric PACT by reconstructing high‐quality images using only 128 US elements. By employing Gabor filter‐based degradation, efficient channel attention, efficient self‐attention, and Mamba, HD‐PACT better renders structural and functional details across different planes, organisms, and wavelengths, all with remarkable time‐efficiency. Its ability to leverage a pretrained model enables dynamic multiparametric imaging for different subsampling settings, even with limited data and training time. We expect that our HD‐PACT will deliver cost‐effective and advanced improvements in PACT imaging, significantly broadening its applicability in both preclinical and clinical settings where resources are limited.

## 4 Experimental Section

### Data Preparation

The described HD‐PACT system was based on a 1024‐element hemispherical transducer array (Japan Probe, Japan) with a 2.02 MHz center frequency and 54% bandwidth. The PA‐excitation laser was a 20 Hz OPO laser (PhotoSonus M‐20, Ekspla, Lithuania), and DAQ was performed by a 256‐channel TX/RX programmable ultrasound imaging system (Vantage 256, Verasonics, USA). All 1024 transducer elements were used to acquire so‐called “1024‐full” PA data. Because this acquisition requires a 4:1 MUX due to the low number of DAQ channels, the frame rate was 5 Hz. Assuming that the 1024 elements were numbered from 1st to 1024th (Figure S2a, Supporting Information), so‐called “256‐sparse” images were reconstructed by using data from the “4i+1”‐th elements of the array (where i ranges from 0 to 255), and “128‐cluster” images were reconstructed with data from the 1st to the 128th elements. In the case of 128‐cluster elements, the DAQ could simultaneously acquire data from 128‐cluster elements. As a result, a frame rate of 20 Hz was achieved, matching the laser's pulse repetition rate. The image reconstruction uses the delay‐and‐sum algorithm, and a whole‐body image was created by stitching together individual volumetric images obtained from each scan position. The details of the developed PACT system was described in the previous studies.^[^ ^50^, ^51^ ^]^

For the training dataset, 54912 sliced 2D PACT images from 6 whole‐body rats (3 ventral‐ and 3 dorsal‐plane) were used. All rat training data were acquired at a wavelength of 900 nm, with each rat imaged in ≈6000 to 16000 2D slices. Detailed information for each rat is presented in Table S1 (Supporting Information). For validation, data from various planes, wavelengths, and organisms were utilized, including ventral and dorsal‐plane whole‐body rat data at 900 nm, sagittal‐plane whole‐body and tumor‐bearing mouse data at multiple wavelengths (730, 756796, and 866 nm), human palm data at 800 nm, and tumor‐bearing mouse data at multiple wavelengths (730, 756, 796, and 866 nm). Both the training and test datasets were normalized to the maximum and minimum values of the whole‐body data. For transfer learning, one whole‐body rat volume and six organ‐specific volumes were utilized for each of the 128‐cluster and 256‐cluster datasets.

### Animal and Human Palm Data Preparation

The animal and human palm datasets utilized in this study were obtained from previous research.^[^ ^50^ ^]^ All animal experiments were conducted in accordance with the regulations and protocols approved by the Institutional Animal Care and Use Committee (IACUC) of Pohang University of Science and Technology (POSTECH, approval No. POSTECH‐2022‐0043 and No. POSTECH‐2023‐0015). Human palm imaging experiments were performed under a clinical protocol approved by the Institutional Review Board (IRB) of POSTECH (PIRB‐2022‐E‐26). One healthy volunteer participated in the study after receiving a full explanation of the procedures and providing written informed consent. Both the operator and the subject wore laser safety goggles and flame‐retardant clothing to ensure protection from laser exposure during the imaging sessions. Further details of the experimental setup and imaging procedures are described in the previous study.^[^ ^50^ ^]^

### Forward Process

There were many degradation operators to invert arbitrary image transforms from high to low quality. The forward process from high to low quality should not be limited to Gaussian noise, as medical‐imaging modalities can contain different types of noise (e.g., blur, masking, and snow). Bansal et al.^[^ ^63^ ^]^ demonstrated that various degradation functions could be used for inverting arbitrary image transforms. For example, CoreDiff,^[^ ^53^ ^]^ one of the generalized diffusion models, designed a CT (Computed Tomography) forward process to properly inject noise into high‐dose CT images. In this study, a Gabor filter was used to reflect the radial‐streaky noise observed in a low‐specification PACT system. The Gabor filter was a linear filter formed by modulating a sine wave with a Gaussian envelope, effectively acting as a bandpass filter for spatial frequencies. A Gabor filter *G*_(*i*,*j*)_ was tuned by parameters that could be flexibly adapted to different features in the image:

(1) $$G_{\left(i,j\right)}\left(\theta ,\lambda ,\sigma ,\psi ,\gamma \right)=\exp\left(-\frac{{i^{\prime }}^{2}+\gamma^{2}{j^{\prime }}^{2}}{2\sigma^{2}}\right)\cdot \cos\left(2\pi \frac{i^{\prime }}{\lambda }+\psi \right)\;$$

where θ, λ, σ, ψ, and γ respectively stand for the filter orientation, wavelength of the sinusoidal wave, standard deviation of the Gaussian function, phase offset of the sinusoidal wave, and the parameter that controls the ellipticity of the Gaussian function. In addition, *i*′ =  *i*cos θ + *j*sin θ;  *j*′ =   − *i*sin θ + *j*cos θ; and (*i*, *j*) is a coordinate.^[^ ^64^ ^]^ Using the Gabor filter *G*, the PACT forward process *D* was defined as follows.

(2) $$\begin{array}{c} D\left(x_{0},x_{T},t\right)\\ =G_{t}\;x_{0}+\left(1-G_{t}\right)x_{T}\\ =\alpha_{t}\;G_{\left(i,j\right)}\left(\theta ,\lambda ,\sigma ,\psi ,\gamma \right)\cdot x_{0}+\left(1-\alpha_{t}G_{\left(i,j\right)}\left(\theta ,\lambda ,\sigma ,\psi ,\gamma \right)\right)\cdot x_{T}\\ =x_{t} \end{array}$$

where *x*_0_ stands for a 1024‐full image and *x*_*T*_ stands for a low‐quality PACT image (a 256‐sparse or 128‐cluster image). The parameter α_*t*_ is a scaling factor that determines the balance between the *x*_0_ and *x_T_* at a given time step (α_*t*_ < α_*t* − 1_,  ∀ 1 ≤ *t* ≤ *T*). The degraded image *x_t_* at time step *t* is obtained through the PACT forward process as *D*(*x*_0_,*x_T_*,*t*).

### Objective Function

The HD model used the two‐stage training approach of CoreDiff^[^ ^53^ ^]^ to enhance the quality of the low‐specification PACT. In stage I, it was assumed that *x_t_*, $x_{T}^{-1}$, and $x_{T}^{+1}$ $\in R^{1\times H\times W}$ are the current, the upper, and the lower slice, respectively. A single 2D slice loses structural details due to noise during the forward process, thus, data were concatenated as $x_{t}^{c}=Concat\left(x_{T}^{-1},\;x_{t},x_{T}^{+1}\right)\in R^{3\times H\times W}$ to get contextual 3D information. Since the adjacent slices share similar structures that were not changed along the *z*‐axis by noise, $x_{t}^{c}$ is used as an input data to provide information that distinguishes between noise and structural detail.^[^ ^65^ ^]^ Given $x_{t}^{c}$, the reverse process *R*_θ_ predicts ${\hat{x}}_{0}$ to approximate *x*_0_:

(3) $${\hat{x}}_{0}=R_{\theta }\left(x_{t}^{c},t\right)$$

(4) $$F_{stage\_I}=\left\|{\hat{x}}_{0}-x_{0}\right\|$$

In stage II, given ${\hat{x}}_{t-1}=D\left({\hat{x}}_{0},x_{T},t-1\right)\in R^{1\times H\times W}$, the HD model receives an input data ${\hat{x}}_{t-1}^{c}=Concat\left(x_{T}^{-1},\;{\hat{x}}_{t-1},\;x_{T}^{+1}\right)\in R^{3\times H\times W}$ passing through an error‐modulated module (EMM). Because the characteristics of the image were subject to change at each time step *t*, it was necessary for the model to reflect features at each time step. In stage I, the model demanded *x_t_* as input data and utilized a multi‐layer perceptron (MLP) to extract time‐embedding vectors at time‐step *t*. However, stage II requires ${\hat{x}}_{t-1}$ as input data, which is different from *x*_*t* − 1_ because ${\hat{x}}_{t-1}$ is created from ${\hat{x}}_{0}$. This difference can lead to misalignment between the time‐embedding at *t* − 1 and ${\hat{x}}_{t-1}$. The EMM corrects the misalignment by using feature‐wise linear modulation with a shallow network *F*_φ_.^[^ ^66^ ^]^ By using ${\hat{x}}_{0}$ and *x_T_*, the shallow network outputs modulation factors β_*t* − 1_ and γ_*t* − 1_ in Equation (5). The t − 1 time embedding vectors *f*_*t* − 1_ are calibrated as Equation (6).

(5) $$\beta_{t-1},\gamma_{t-1}=F_{\varphi }\left({\hat{x}}_{0},\;x_{T}\right)$$

(6) $${\tilde{f}}_{t-1}=\gamma_{t-1}\;f_{t-1}+\beta_{t-1},\;f_{t-1}=MLP\left(SinPE\left(t-1\right)\right)$$

In stage II, the reverse process $R_{\theta }\left({\hat{x}}_{t-1}^{c},t-1,F_{s}\left({\hat{x}}_{0},\;x_{T}\right)\right)$ outputs ${\hat{\hat{x}}}_{0,}$ and the $F_{stage\_\mathrm{II}}$ loss as follows:

(7) $${\hat{\hat{x}}}_{0}=R_{\theta }\;\left({\hat{x}}_{t-1}^{c},t-1,F_{\varphi }\left({\hat{x}}_{0},\;x_{T}\right)\right)$$

(8) $$F_{stage\_\mathrm{II}}=\left\|{\hat{\hat{x}}}_{0}-x_{0}\right\|\;$$

As mentioned in Note S1 (Supporting Information), the generalized diffusion‐based model still introduces an error at the *t* − 1 step, so the error loss *F_error_* was designated as follows:

(9) $$F_{error}=\left\|{\hat{x}}_{t-1}-x_{t-1}\right\|$$

The final loss function of the HD model was calculated by Equation (10), and the detailed training procedure is described in Note S2 (Supporting Information):

(10) $$L_{final}=F_{stage\_I}+F_{stage\_\mathrm{II}}+F_{error\;}$$

### Sampling Process

The HD model follows an improved sampling strategy for the generalized diffusion. *R*_θ_ cannot perfectly restore the original high‐quality image *x*_0_, thereby iteratively accumulating error and causing the inaccurate degraded result. By taking additional degraded data at each *t* − 1 step during the sampling, the HD model yields lower error than naïve sampling, as shown in Note S1 (Supporting Information). First, the input image $x_{T}^{C}$ is generated by concatenating the low‐quality PACT slice *x_T_* image with its adjacent slices $x_{T}^{-1}$ and $x_{T}^{+1}$. In stage I, the model outputs ${\hat{x}}_{0}$ and the forward process generates ${\hat{x}}_{t-1}$ (Equations (11) and (12) and Note S1, Supporting Information). In stage II, ${\hat{x}}_{t-1}^{c}$ is formed by concatenating three slices ($x_{T}^{-1},\;{\hat{x}}_{t-1},x_{T}^{+1}$), thus allowing the model to estimate ${\hat{\hat{x}}}_{0}$. The detailed sampling algorithm is described in Note S3 (Supporting Information).

(11) $${\hat{x}}_{t}=\left(x_{t}-G_{t}{\hat{x}}_{0}\right)\;/\left(1-G_{t}\right)$$

(12) $${\hat{x}}_{t-1}=x_{t}\;-D\left({\hat{x}}_{0},{\hat{x}}_{t},t\right)+D\left({\hat{x}}_{0},{\hat{x}}_{t},t-1\right)$$

### Network

The HD model was designed with EHM blocks (Figure 2c). The input data is denoted as *f* ^1 × *X* × *Y*^. After a convolutional filter extracts the local features *f* ^*C* × *X* × *Y*^, the EHM block outputs comprehensive features *f* ^*C* × *X* × *Y*^ through four steps. In the first step, the efficient channel attention (ECA) module extracts feature map $f_{E}^{C\times X\times Y}$, considering local cross‐channel interaction:^[^ ^54^ ^]^

(13) $$\omega_{i}=\sigma \left(\sum_{j=1}^{k}w^{j}z_{i+j}\right)$$

where global average pooling g(*f* ^*C* × *X* × *Y*^) outputs *z*_*i* + *j*_ ∈ *f^C^* and ω_*i*_ means an attention value to be applied to the i‐th channel. $\mathrm{Then},\;f_{E}^{C\times X\times Y}$ is calculated as

(14) $$f_{E}^{C_{i}\times X\times Y}=f^{C_{i}\times X\times Y}\;\cdot \omega_{i}$$

Second, the EHM block generates query, key, and value feature maps by reducing the key and the value. This efficient self‐attention strategy not only reduces the computational complexity through the subsampling, but also improves performance because the attention score map has a low‐rank property.^[^ ^47^, ^67^ ^]^ Query *f_Q_*, Key $f_{\hat{K}}$, and value $f_{\hat{V}}$ map are then computed as follows:

(15) $$f_{Q}^{C\times X\times Y}=f_{E}W^{Q}\;,\;\;f_{K}^{C\times X\times Y}=f_{E}W^{K}\;,f_{Q}^{C\times X\times Y}=f_{E}W^{V}$$

(16) $$f_{\hat{K},\hat{V}}^{C\times x\times y}=f_{K,V}^{C\times \frac{X}{r}\times \frac{Y}{r}}\;$$

where *r* is a reduction ratio. Third, the EHM block refines the key and the value by a Mamba, an extension of state space sequence models (SSMs). SSMs is a system of linear ordinary differential equations (ODEs) that predicts future behavior *y*(*t*) based on input u(t)∈R and the current or hidden state $x\left(t\right)\in R^{N}$. SSMs can be expressed by state space equations consisting of a state Equation (17) and an output equation (18):

(17) $$\dot{x}\;\left(t\right)=Ax\left(t\right)+Bu\left(t\right)$$

(18) $$y\;\left(t\right)=Cx\left(t\right)$$

Here $A\in R^{N\times N}$ represents a dynamic property of the state system, $B\in R^{N\times N}$ is a matrix representing the effect of inputs on state change, and $C\in R^{N\times N}$ is a matrix that describes how the current state *x*(*t*) affects the output *y*(*t*). Since an SSM is defined on a continuous system, a discretization method called a zero‐order hold (ZOH) is required to apply SSMs on digital systems such as computers. The discretization rule can be stated as follows:

(19) $$\bar{A}=\;\exp\left(\Delta A\right)$$

(20) $$\bar{B}={\left(\Delta A\right)}^{-1}\;\left(\exp\left(\Delta A\right)-I\right)\cdot \Delta B$$

Discrete SSMs can calculate linear recurrence (Equation 22) as global convolution (Equation 24):

(21) $$x\;\left(t\right)=\bar{A}\;x\left(t-1\right)+\bar{B}u\left(t\right)$$

(22) $$y\;\left(t\right)=Cx\left(t\right)$$

(23) $$\bar{K}=\left(C\bar{B},\;C\bar{AB},\;\text{…},C{\bar{A}}^{L-1}\bar{B}\right)$$

(24) $$SSM\;\left(u\right)=u*\bar{K}$$

where *L* denotes the length of the input sequence. The Mamba with SSMs algorithm is simply described in Note S4 (Supporting Information). Mamba outputs feature maps $f_{\hat{K},\hat{V}}^{C\times x\times y}$ of the refined key and value (Figure 2c), and the EHM block creates the final feature map,

(25) $$\mathrm{softmax}\left(\frac{f_{Q}{f_{\hat{K}}}^{T}}{\sqrt{C^{3}}}\right)f_{\hat{V}}$$

### Training and Implementation Details

In the PA forward process of our HD model, the parameters of the Gabor filter include an orientation θ of π/4, a wavelength λ of 3.3, a Gaussian standard deviation σ of 10, a phase offset ψ of 0, and an ellipticity control parameter γ of 1. Further,  α_*t*_ is scaled linearly from 0.999 to 0 as it varies from α_1_,  · · ·,  α_*T*_ (Equation 2). In the training process of the 256‐sparse data, the HD model was trained using a mini‐batch size of 4, a learning rate of 2 × 10^−4^, the Adam optimizer with b1 = 0.9 to b2 = 0.99, and a total of 125K iterations. The HD model used an input size of 3 × 128 × 128 with three adjacent slices, and a time step *T* = 2. During the transfer learning phase, all training details were the same as in the 256‐sparse training, except for the total number of training iterations. To demonstrate the performance of our transfer model with a small amount of training data and less fine‐tuning time, 9792 iterations were applied for the transfer learning on 128‐cluster data. For the comparison, a single Nvidia GeForce RTX 3090 GPU with 24 GB memory was used for both training and testing. The training and testing processes were conducted using Python version 3.10.13 and the PyTorch framework version 2.2.1 on a Linux system. All hyperparameters for each DL model followed the corresponding original paper and official source codes.

### Evaluation Metrics

To evaluate the structural quality of images, PSNR and SSIM were used to quantify the similarity between a prediction *x* and ground truth *y*. Higher PSNR and SSIM represent better performance. Based on the mean square error (MSE), PSNR is defined as

(26) $$PSNR\;\left(x,\;y\right)=10\log_{10}\left(\frac{MAX^{2}}{MSE\left(x,\;y\right)}\right)$$

where MAX denotes the range of the data type, which goes from 0 to 1 in the dataset. SSIM was a metric reflecting how humans perceive images with respect to the three main types of visual information—luminance, contrast, and structural information—and it is defined as

(27) $$SSIM\;\left(x,\;y\right)=\frac{\left(2\mu_{x}\mu_{y}+C_{1}\right)\left(2\sigma_{x}\sigma_{y}+C_{2}\right)}{\left(\mu_{x}^{2}+\mu_{y}^{2}+C_{1}\right)\left(\sigma_{x}^{2}+\sigma_{y}^{2}+C_{2}\right)},$$

where μ_*x*_ and σ_*x*_ are respectively the mean and variance of *x*, and σ_*xy*_ is the covariance of *x* and *y*. All statistics calculated from experimental results are expressed as mean ± standard deviation (s.d.), and statistical significance between two groups was determined using a paired *t*‐test.

PA functional parameters, including HbT and sO_2,_ were calculated through spectral unmixing based on the linear least‐squares fitting (LSQ) method.^[^ ^50^, ^51^ ^]^ PA images were obtained using four‐wavelength data at 730, 756, 796, and 866 nm. Before spectral unmixing, each reconstructed PA image was smoothened using a median filter with size of 3 × 3 × 3 voxels and normalized by the measured laser power. Then, LSQ unmixing was applied to every voxel as follows, using the known extinction coefficients of HbO and HbR:

(28) $$\left[\begin{array}{c} \begin{array}{cc} \varepsilon_{HbO}^{730} & \varepsilon_{HbR}^{730}\\ \varepsilon_{HbO}^{756} & \varepsilon_{HbR}^{756} \end{array}\\ \begin{array}{cc} \varepsilon_{HbO}^{796} & \varepsilon_{HbR}^{796}\\ \varepsilon_{HbO}^{866} & \varepsilon_{HbR}^{866} \end{array} \end{array}\right]\;\left[\begin{array}{c} C_{HbO}\\ C_{HbR} \end{array}\right]=\left[\begin{array}{c} \begin{array}{c} PA_{730}\\ PA_{756} \end{array}\\ \begin{array}{c} PA_{796}\\ PA_{866} \end{array} \end{array}\right]\;$$

where ε is the molar extinction coefficient for each wavelength, C is the concentration of either HbO or HbR, and PA is the PA‐voxel value. With the unmixed concentrations of the HbO and HbR values, the sO_2_ and concentration of HbT were calculated as follows:

(29) $$sO_{2}=\frac{C_{\mathrm{HbO}}}{C_{\mathrm{HbO}}+C_{\mathrm{HbR}}},\;\;C_{\mathrm{HbT}}=C_{\mathrm{HbO}}+C_{\mathrm{HbR}}\;$$

To quantify the HbT and vessel density of the tumor, the tumor region was manually segmented based on the HbT image. For the selected tumor ROI, thresholding was applied to segment the vascular network. The vessel density was calculated by dividing the vessel area by the total area of the ROI, and the HbT level in the tumor region was expressed as the mean HbT value among the ROI vessel voxels.

## Conflict of Interest

Corresponding author Chulhong Kim has financial interests in OPTICHO, which, however, did not support this work.

## Author Contributions

H.J., S.O., and S.C. contributed equally to this work. H.J., S.O., and S.C planned the study and drafted the manuscript. S.C., J.K and J.Y prepared the data. H.J. and S.O designed and carried out the main framework. C.K. supervised the project. All authors discussed the results and contributed to the writing.

## Supporting information



Supporting Information

Supplementary Video 1

Supplementary Video 2

Supplementary Video 3

Supplementary Video 4

Supplementary Video 5

Supplementary Video 6

## Data Availability

The data that support the findings of this study are available from the corresponding author upon reasonable request.
