# Supplementary material for: A Hybrid Diffusion Model Enhances Multiparametric 3D Photoacoustic Computed Tomography
Source: Adv Sci (Weinh). 2025 Oct 23;13(1):e13624. doi: 10.1002/advs.202513624 (PMC12767131; doi:10.1002/advs.202513624)
Supplement: Supplementary file 1 — Supporting Information [file ADVS-13-e13624-s002.docx]

**Supporting Information for**

**A Hybrid Diffusion Model Enhances Multiparametric 3D Photoacoustic Computed Tomography**

*Hyunsu Jeong^1^, †, Seunghun Oh^1^, †, Seongwook Choi^2^, †, Jiwoong Kim^1^, Jinge Yang^3^, and Chulhong Kim^1^**

^1^Graduate School of Artificial Intelligence, Departments of Electrical Engineering, Convergence IT Engineering, Mechanical Engineering, Medical Science and Engineering, and Medical Device Innovation Center, Pohang University of Science and Technology (POSTECH), Pohang, Republic of Korea

^2^Department of Radiology, Molecular Imaging Program at Stanford (MIPS), School of Medicine, Stanford University, California, USA

^3^Departments of Medical Engineering, California Institute of Technology, California, USA

These authors contributed equally: Hyunsu Jeong, Seunghun Oh, Seongwook Choi

*Corresponding author: Chulhong Kim (chulhong@postech.edu)

**Table of contents**

**Supplementary Notes**

1. The improved sampling of a generalized diffusion.

2. HD-PACT training algorithm.

3. HD-PACT sampling algorithm.

4. Mamba algorithm.

**Supplementary Figures**

1. Schematic of the PACT imaging system.

2. Comparison of hemispherical US transducer configurations and their imaging performances.

3. Visualization performance of models with various forward operators.

4. Comparison of inference speeds and attention module in the HD model.

5. Depth-encoded whole-body rat PACT images captured in the ventral plane at 900 nm.

6. Whole-body mouse MAP images captured in the sagittal plane at 730 nm.

7. Qualitative comparison of all denoising methods on the sagittal whole-body mouse dataset at 730 nm and the human palm dataset at 800 nm.

8. sO₂ distribution in a B-mode section image of a tumor volume at day 12.

9. Power spectrum of rat heart PA signals using 128-cluster elements.

10. Dynamic results using transfer learning for a 256-cluster dataset.

11. Maximum-amplitude projections of the human palm vessel region and spatiotemporal resolution across imaging modes (full width at half maximum (FWHM)).

12. Comparison of difference maps between intermediate states along the forward process ($T=2$ and $T=1000$).

13. Comparison of SNR and CNR according to depth and organ.

14. Comparison of SNR and CNR at different signal levels in tumor-induced angiogenesis.

**Supplementary Tables**

1. Data arrangement for 256-sparse experiments.

2. Comparative performances of three key components in the Efficient Hybrid Module on the 256-sparse elements dataset.

3. Quantitative comparison of all denoising methods on the entire 256-sparse test dataset.

4. Quantitative comparison of all denoising methods on the sagittal whole-body mouse dataset at 730 nm and the human palm dataset at 800 nm.

5. Quantitative comparison of the transfer HD model with the scratch HD model across individual 128-cluster elements datasets.

**Supplementary Videos**

1. Time-lapse PA sO2 MAP images of a rat’s brain under an oxygen challenge, reconstructed from a 128-cluster element dataset, as the prediction of the transfer HD model, and from a 1024-full elements dataset.

2. Time-lapse PA MAP images of a rat’s kidney, reconstructed from a 128-cluster elements dataset, as the prediction of the transfer HD model, the prediction of the scratch HD model, and from a1024-full elements dataset.

3. Time-lapse PA MAP images of a rat’s heart captured at 20 Hz, reconstructed from a 128-cluster elements dataset, as the prediction of the transfer HD model, the prediction of the scratch HD model, and at 5 Hz using a 1024-full elements dataset.

4. Time-lapse PA sO2 MAP images of a rat’s brain under an oxygen challenge, reconstructed from a 256-cluster elements dataset, as the prediction of the transfer HD model, and from a 1024-full elements dataset.

5. Time-lapse PA MAP images of a rat’s kidney, reconstructed from a 256-cluster elements dataset, as the prediction of the transfer HD model, the prediction of the scratch HD model, and from a 1024-full elements dataset.

6. Time-lapse PA MAP images of a rat’s heart at 20 Hz, reconstructed from a 256-cluster elements dataset, as the prediction of the transfer HD model, the prediction of the scratch HD model, and at 5 Hz using a 1024-full elements dataset.

**Supplementary Notes**

**Note S1.** **Improved sampling of a generalized diffusion.**

The naïve sampling of denoising diffusion implicit model (DDIM) is expressed by Algorithm 1. After the reverse process $R_{\theta}$ and the forward process $D have been iterated$, the degraded image $x_{t}$ can be approximately restored to $\hat{x}_{0}\approx x_{0}$.

| **Algorithm 1** Naïve Sampling in DDIM |
| --- |
| **Input:** A Gaussian-noised sample $x_{t}$  **for** $t=T, T-1, \ldots, 1$ **do**  $\hat{x}_{0}\leftarrow R_{\theta}\left( x_{t},t \right)$  $x_{t-1}=D(\hat{x}_{0}, t-1)$  **End for**  **Return:** $x_{0}$ |

Algorithm 1 shows that the diffusion model causes iterative error because of the imperfect $R$ and $D$. Given $X_{t}=\sum_{i=t}^{T} x_{i}=D(x_{0},t)$ and $\hat{X}_{t}=\sum_{i=t}^{T} \hat{x}_{i}=D(\hat{x}_{0},t)$, the error $E$ can be defined as $\left\| X_{t-1}-\hat{X}_{t-1} \right\|$.

| $E_{t}$  $=\left\Vert X_{t-1}-\hat{X}_{t-1} \right\Vert$  $=\left\Vert\sum_{i=t-1}^{T} x_{i} -\sum_{i=t-1}^{T} \hat{x}_{i} \right\Vert$  $=\left\Vert\sum_{i=t-1}^{T} \left( x_{i}-\hat{x}_{i} \right) \right\Vert$  $=\sum_{i=t-1}^{T} \left\Vert x_{i}-\hat{x}_{i} \right\Vert$. |
| --- |

To reduce the errors, a cold diffusion applies a first-order approximation in the Taylor series on $D\left( x,t \right)$ near $x_{0}$ and $t$=0. By expanding up to the first-order term for each variable, the changes in $x$ and $t$ are considered separately. As the goal is to model the process of change$\mathrm{only}\mathrm{for}$ step *t*, only the term involving $t$ is handled. The first-order Taylor polynomial derives the degradation $D\left( x_{0},t \right)\approx x_{0}+t\cdot e$.

| $D\left( x_{0},t \right)$  $=D\left( x_{0},0 \right)+\left. \frac{\partial D}{\partial x} \right\vert_{(x_{0},0)}\left( x-x_{0} \right)+\left. \frac{\partial D}{\partial t} \right\vert_{(x_{0},0)}t+$high-order terms  $\approx D\left( x_{0},0 \right)+\left. \frac{\partial D}{\partial t} \right\vert_{(x_{0},0)}t$  $=x_{0}+\left. \frac{\partial D}{\partial t} \right\vert_{(x_{0},0)}t$  $=x_{0}+t\cdot e .$ |
| --- |

Through a multivariate Taylor expansion, the first-order Taylor polynomial of the forward process $x_{t-1}$ at $t-1$ step can be defined as follows.

| $x_{t-1}$  $=D\left( x_{0},t-1 \right)$  ${=x}_{0}+(t-1)\cdot e$  $=x_{0}+t\cdot e-R\left( x_{t},t \right)-t\cdot e+R\left( x_{t},t \right)+(t-1)\cdot e$  $\approx D\left( x_{0},t \right)-D\left( R\left( x_{t},t \right),t \right)+D\left( R\left( x_{t},t \right),t-1 \right)$  $=x_{t}-D\left( R\left( x_{t},t \right),t \right)+D\left( R\left( x_{t},t \right),t-1 \right).$ |
| --- |

Based on the redefined forward process $x_{t-1}$, the sampling process of the generalized diffusion is newly defined as follows.

| **Algorithm 2** Improved Sampling |
| --- |
| **Input:** A Gaussian noised sample $x_{t}$  **for** $t=T, T-1, \ldots, 1$ **do**  $\hat{x}_{0}\leftarrow R_{\theta}\left( x_{t},t \right)$  $x_{t-1}=x_{t}-D\left( \hat{x}_{0}, t \right)+D\left( \hat{x}_{0}, t-1 \right)$  **End for**  **Return:** $x_{0}$ |

The improved sampling approach reduces the error caused by the imperfect $R_{\theta}$ because only the error at the $t-1$ step affects the model. Given $X_{t}=\sum_{i=t}^{T} x_{t}=D(x_{0},t)$ and $\hat{X}_{t}=\sum_{i=t}^{T} \hat{x}_{t}=x_{t}-D\left( \hat{x}_{0}, t \right)+D\left( \hat{x}_{0}, t-1 \right)$, the error $E$ can be defined as $\left\| X_{t-1}-\hat{X}_{t-1} \right\|$. We can see that the improved sampling strategy produces less error as follows:

| $E_{t}$  $=\left\Vert X_{t-1}-\left( X_{t}-D\left( \hat{X}_{0},t \right)+D\left( \hat{X}_{0},t-1 \right) \right) \right\Vert$  $=\left\Vert\left( D\left( \hat{X}_{0},t \right)-D\left( \hat{X}_{0},t-1 \right) \right)-\left( X_{t}-X_{t-1} \right) \right\Vert$  $=\left\Vert\left( \sum_{i=t}^{T} \hat{x}_{i}-\sum_{i=t-1}^{T} \hat{x}_{i} \right)-\left( \sum_{i=t}^{T} x_{i}-\sum_{i=t-1}^{T} x_{i} \right) \right\Vert$  $= \left\Vert x_{t-1}-\hat{x}_{t-1} \right\Vert.$ |
| --- |

The loss function of the generalized diffusion can be briefly defined as follows:

| $L_{\theta}=\min_{\theta} \mathbb{E}_{x_{0}\sim Q,x_{0}\sim P}\left\Vert R_{\theta}\left( D(x_{0},t \right),t)-x_{0} \right\Vert.$ |
| --- |

**Note S2.** **HD-PACT training algorithm.**

The model training procedure, shown in Algorithm 3, involves two stages. In stage Ⅰ, a degraded PACT image $x_{t}$ is generated by the degradation operator $D$ from a high-quality PACT image $x_{0}$ (1024-full image), a low-quality PACT image $x_{T}$ (e.g., a 256-sparse or a 128-cluster image), and the total time step *T*, which is randomly sampled among 1,…,T. Then, $x_{t}\in\mathbb{R}^{1\times H\times W}$ is concatenated with adjacent low-quality images $x_{T}^{-1}$ and $x_{T}^{+1}$, making concatenation data $x_{t}^{c}\in\mathbb{R}^{3\times H\times W}$. At time step $t$, $x_{t}^{c}$ is utilized as the input of the denoising model $R_{\theta}(.)$ to estimate $\hat{x}_{0}\in\mathbb{R}^{1\times H\times W}$.

In stage Ⅱ, low quality image $x_{T}$ and $\hat{x}_{0}$ are used to create $\hat{x}_{t-1}$, which is then concatenated with neighboring images $x_{T}^{-1}$ and $x_{T}^{+1}$ to predict $\hat{x}_{t-1}^{c}$. However, since there are misalignments between the input data and the corresponding time step $t-1$, the error-modulated module (EMM) adjusts the misalignment using a shallow network $F_{\varphi}$ that provides modulation factors. Finally, $\hat{x}_{t-1}^{c}$, $F_{\varphi}$, and $\hat{x}_{t-1}^{c}$ are used as inputs to predict the ${\hat{\hat{x}}}_{0}$ at time step $t-1$ by the reconstruction model $R_{\theta}$. During training, three loss function terms are minimized: (1) $\left\| \hat{x}_{0}-x_{0} \right\|$, the difference between $x_{0}$ and $\hat{x}_{0}$ calculated in stage Ⅰ, (2) $\left\| {\hat{\hat{x}}}_{0}-x_{0} \right\|$, the difference between $x_{0}$ and ${\hat{\hat{x}}}_{0}$ calculated in stage Ⅱ, and 3) $\left\| \hat{x}_{t-1}-x_{t-1} \right\|$, the difference between $x_{t-1}$ and the degraded image $\hat{x}_{t-1}$ calculated in stage Ⅱ to reduce the remaining error.

| **Algorithm 3** HD-PACT Training |
| --- |
| **Input:** Paired PACT image sets $I= \left\{ \left( x_{0},x_{T} \right)_{i} \right\}_{i=1}^{N}$, total time step *T*, low-quality PACT image $x_{T}$, high-quality PACT image $x_{0}$, the number of slices $N$  **repeat**  Sample *t* ~ Uniform({1,…,T})  $x_{t} \leftarrow D\left( x_{0},x_{T},t \right)$ $\Longrightarrow$ Stage Ⅰ  $x_{t}^{c} \leftarrow Concat(x_{T}^{-1}, x_{t},x_{T}^{+1})$  $\hat{x}_{0} \leftarrow R_{\theta}\left( x_{t}^{c},t \right)$  $\hat{x}_{t-1} \leftarrow$ $D\left( \hat{x}_{0},x_{T},t-1 \right)$ $\Longrightarrow$ Stage Ⅱ  $\hat{x}_{t-1}^{c} \leftarrow Concat\left( x_{T}^{-1}, \hat{x}_{t-1} ,x_{T}^{+1} \right)$  ${\hat{\hat{x}}}_{0} \leftarrow R_{\theta}\left( \hat{x}_{t-1}^{c},t-1,F_{\varphi}(\hat{x}_{0}, x_{T}) \right)$  $L \leftarrow\left\Vert\hat{x}_{0}-x_{0} \right\Vert+\left\Vert{\hat{\hat{x}}}_{0}-x_{0} \right\Vert+\left\Vert\hat{x}_{t-1}-x_{t-1} \right\Vert$  **Update:** $\theta, \varphi$ by $L$  **Until** converged |

**Note S3.** **HD-PACT sampling algorithm.**

In Algorithm 4, the sampling of the HD-PACT conducts the iterative restoration process using the trained denoising model $R_{\theta}$. First, the input low-quality image $x_{T}$ is concatenated with its neighboring slices $x_{T}^{-1}$ and $x_{T}^{+1}$. The restoration model $R_{\theta}$ infers an initial $\hat{x}_{0}$ from the concatenated input $x_{t}^{c}$ at the current time step $t.$ Then, $\hat{x}_{0}$ and $x_{t}$ are used to get the estimated-noisy slice $\hat{x}_{t}$ at the time step $t$. Next, $\hat{x}_{t-1}$ is obtained through the improved sampling. In stage II, the concatenated input $\hat{x}_{t-1}^{c}$ passes through the network while the shallow network, the EMM, calibrates the misalignment at the time step $t-1$. Finally, the HD-PACT outputs an estimated-denoised slice through the two stages.

| **Algorithm 4** HD-PACT Sampling |
| --- |
| **Input:** low-quality PACT image $x_{T}$, total time step *T*, the number of slices $N$  **Output:** Denoised PACT ${\hat{\hat{x}}}_{0}$  $x_{T}^{c} \leftarrow Concat(x_{T}^{-1}, x_{T},x_{T}^{+1})$  **for** $t=T, T-1, \ldots, 1$ **do**  $\hat{x}_{0} \leftarrow R_{\theta}\left( x_{T}^{c},t \right)$ $\Longrightarrow$ Stage 1  $\hat{x}_{t}=\left( x_{t}-G_{t}\hat{x}_{0} \right)/\left( 1-G_{t} \right)$  $\hat{x}_{t-1} \leftarrow$ $x_{t}-D\left( \hat{x}_{0},\hat{x}_{t},t \right)+D\left( \hat{x}_{0},\hat{x}_{t},t-1 \right)$ $\Longrightarrow$ Stage 2  $\hat{x}_{t-1}^{c} \leftarrow Concat\left( x_{T}^{-1}, \hat{x}_{t-1} ,x_{T}^{+1} \right)$  ${\hat{\hat{x}}}_{0} \leftarrow R_{\theta}\left( \hat{x}_{t-1}^{c},t-1,F_{\varphi}(\hat{x}_{0}, x_{T}) \right)$  **end for** |

**Note S4.** **Mamba algorithm.**

Algorithm 5 shows the Mamba process with the selective state spaces model (SSM). The Mamba is designed to process input features $u\in\mathbb{R}^{B\times L\times D}$, where $B$ represents the batch size, $L$ is the input sequence length, and $D$ is the input dimension. This algorithm performs sequential feature transformations using learnable parameters and linear mappings. The three linear projections ($\Delta$, B, C) are computed using the input $u$, activated by the SiLU function. The transformed parameter is calculated as the element-wise exponential with a learnable parameter A and $\Delta$. Another transformed parameter is computed using the matrix inversion of $\left( \Delta A \right)^{-1}$, combined with the exponential operation and a dot product with $\Delta B$. Finally, the output $y$ is generated by the SSM function that takes $\bar{A},\bar{B},C$ and the input feature as arguments.

| **Algorithm 5** Mamba Pseudo-code |
| --- |
| **Input:** the feature $u\in\mathbb{R}^{B\times L\times D}$, batch Size: B, input sequence length: L, input dimension: D  **Params:** A $\leftarrow$ nn.Parameter  **Output:** $y\in\mathbb{R}^{B\times L\times D}$  $\Delta$, B, C = Linear ($SiLU(u)),$ Linear ($SiLU(u)),$ Linear ($SiLU(u))$  $\bar{A}$ = $exp(\Delta A)$  $\bar{B}=\left( \Delta A \right)^{-1}(\exp\left( \Delta A \right)-I)\cdot\Delta B$  $y$ $\leftarrow$ SSM($\bar{A},\bar{B},C)($u) |

**Supplementary Figures**

**
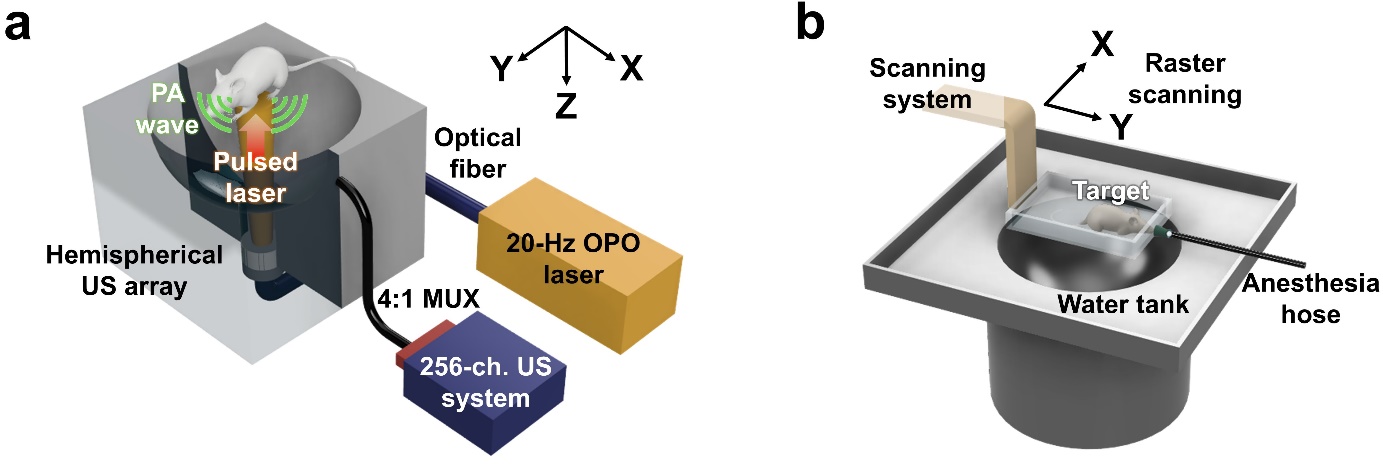
**

Figure S1. Schematic of the PACT imaging system. a) PACT based on a hemispherical ultrasound transducer array. b) Whole-body scanning system.


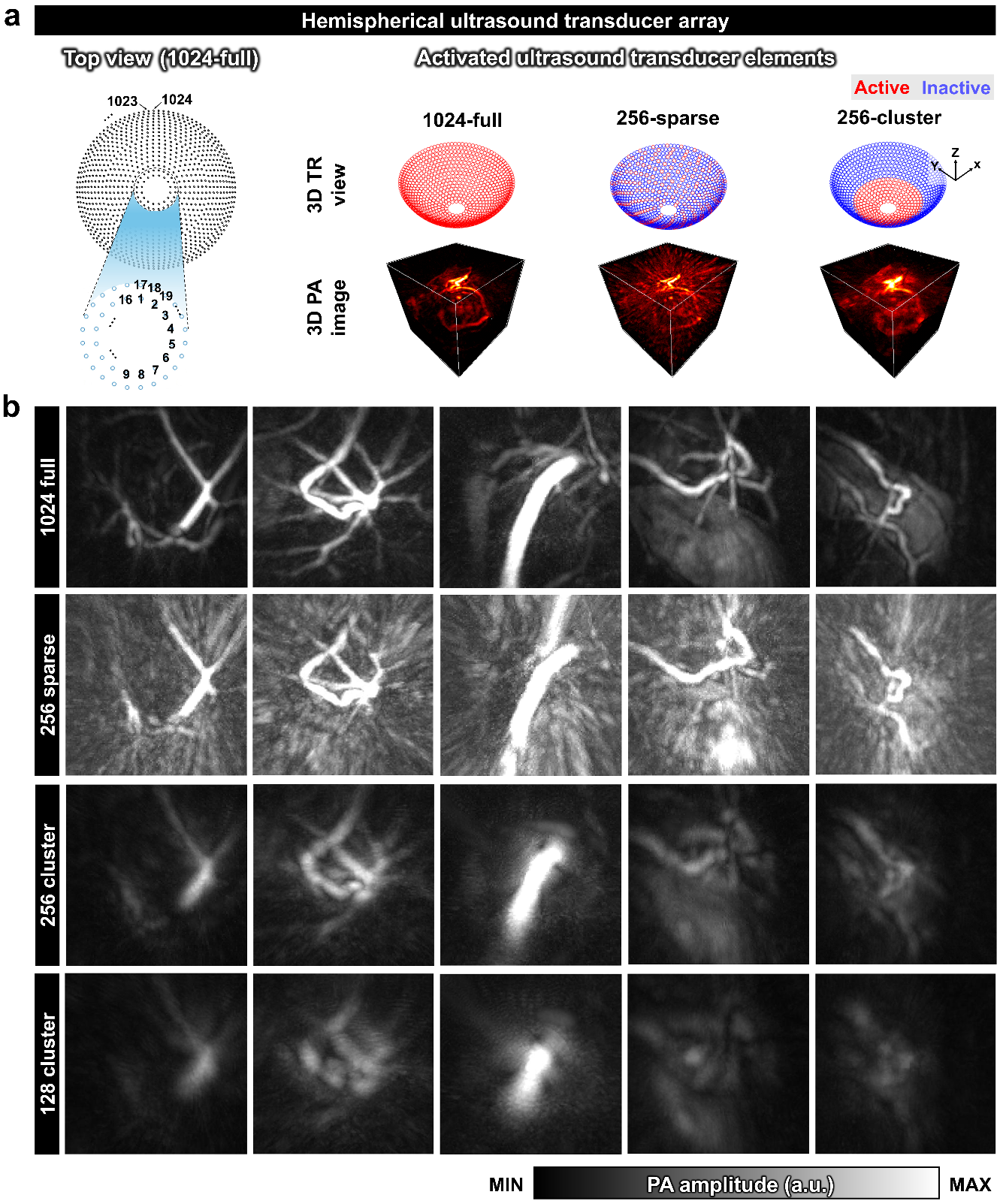


Figure S2. Comparison of hemispherical US transducer configurations and their imaging performances. a) Top view of the 1024-full US transducer elements and a comparison of hemispherical US transducer elements; 1024-full, 256-sparse, and 256-cluster arrays. b) Comparison of single volume maximum amplitude projection (MAP) images: 1024-full, 256-sparse, 256-cluster, and 128-cluster elements.


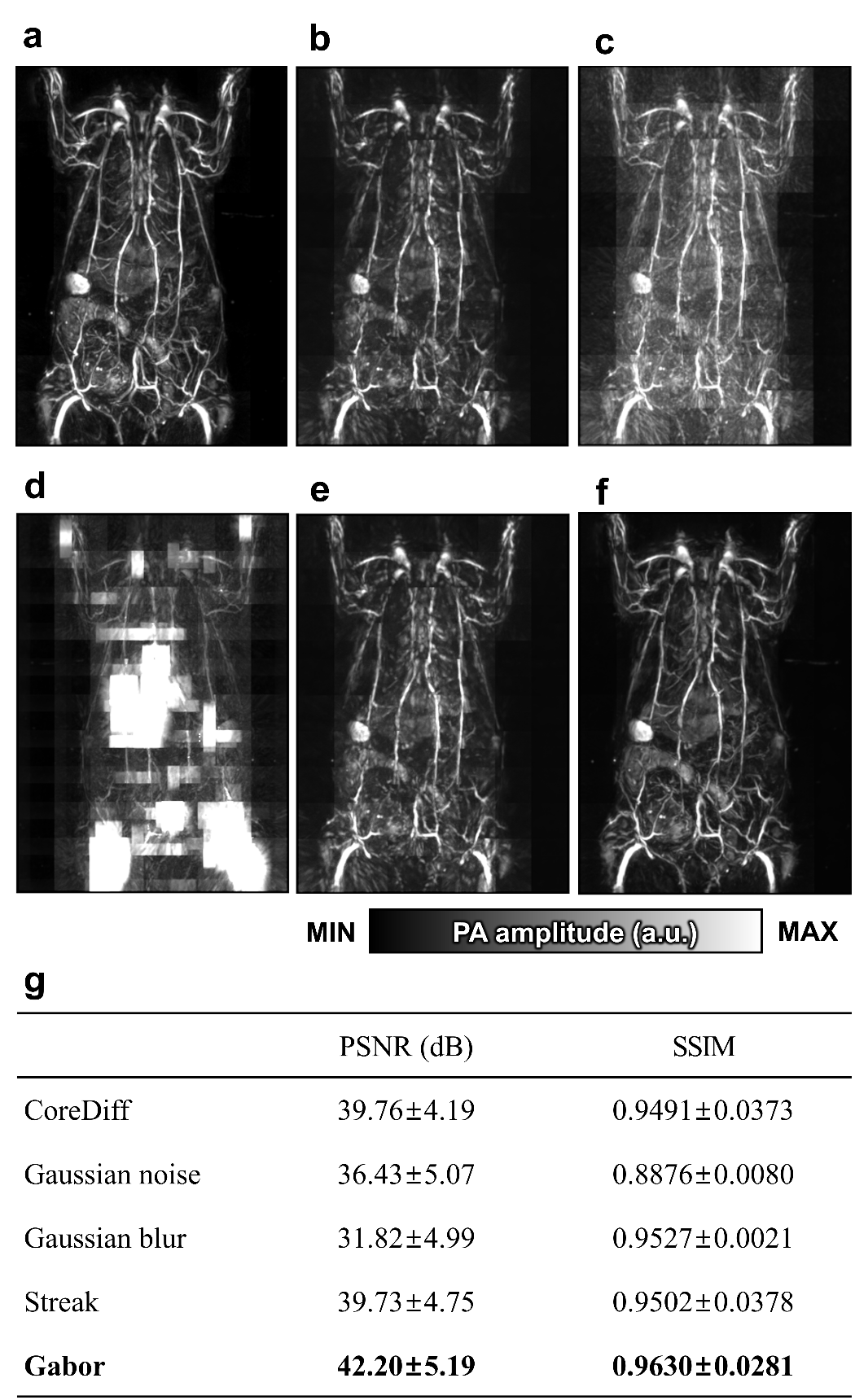


Figure S3. Visualization performance of models with various forward operators. a) PA image of 1024-full elements. b-f) Whole-body rat images inferred using different forward operators, including CoreDiff (b), Gaussian noise (c), Gaussian Blur (d), Streak filter (e), and Gabor filter (f). The images show that the type of forward operator heavily affects the quality of MAP images. g) Quantitative comparison of various forward degradations on a 256-sparse dataset. The performances of models trained with various forward processes are presented. CoreDiff means a degradation operator used in CoreDiff. ^1^ The Gabor filter shows significantly better performance (p<0.001) than the other degradations, including CoreDiff, Gaussian noise, Gaussian blur, and streak, in a paired t-test. The best performances are in bold.


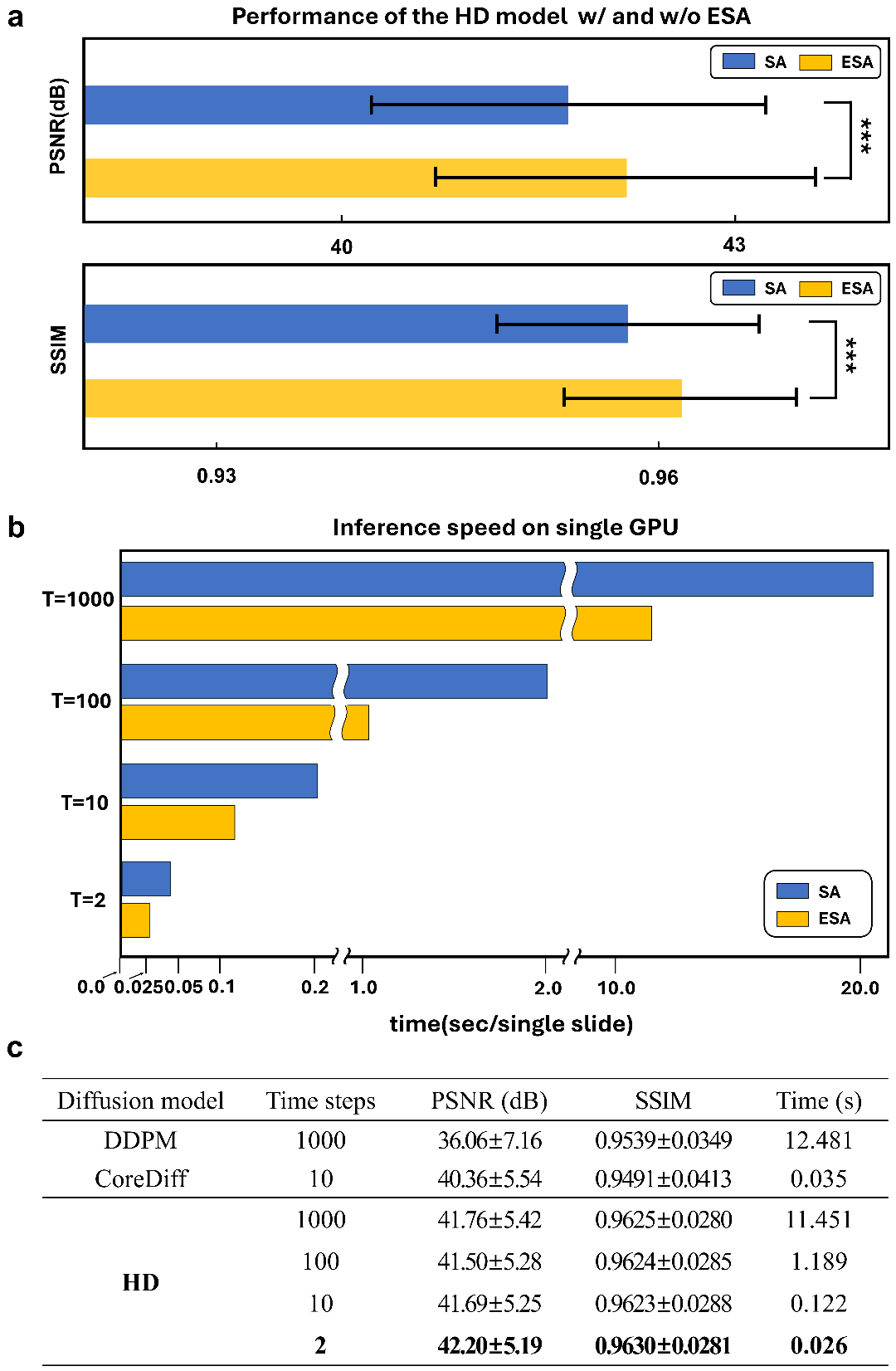


Figure S4. Comparison of inference speeds and attention modules in the HD model. a) Quantitative comparison of the HD model with and without ESA. The HD model outperformed the HD model with SA in terms of PSNR (ESA, 41.70 ± 5.19; SA, 41.37 ± 5.34) and SSIM (ESA, 0.9612 ± 0.0300; SA, 0.9575 ± 0.0331), with statistical significance (p < 0.001). b) Comparison of inference speeds with efficient self-attention (ESA) and with self-attention (SA) by time step. Applying ESA in the HD model achieves an average inference speed approximately 1.8 times faster than SA. c) Comparison of inference times on different-time steps. The HD model demonstrates that the diffusion-based model can greatly reduce the inference time without compromising performance. Compared to more than two time steps, two time steps shows better performance and faster speed in a paired t-test (p<0.001). Furthermore, when compared with other diffusion-based models, the HD model achieves a shorter inference time while maintaining better quantitative performance (p<0.001). The best performances are in bold.


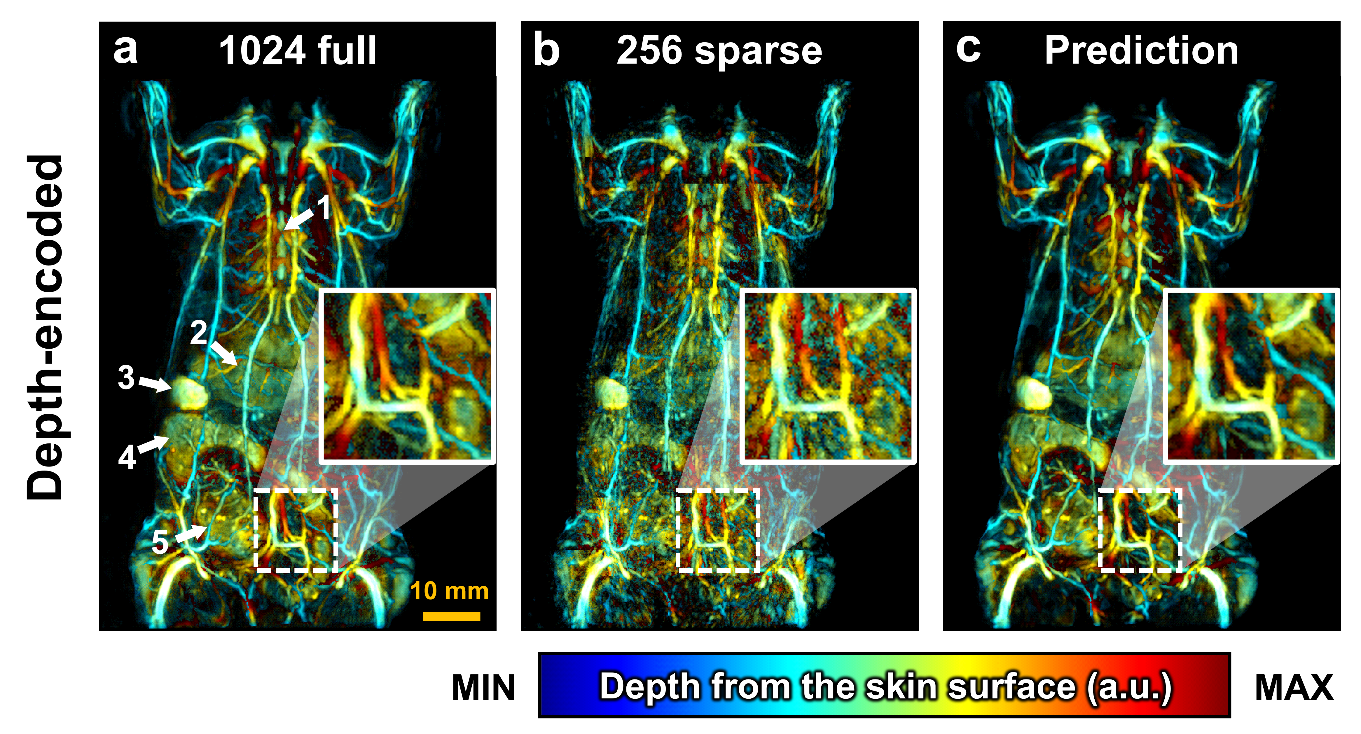


Figure S5. Depth-encoded whole-body rat PACT images captured in the ventral plane at 900 nm. a) 1024-full elements. b) 256-sparse elements. c) HD prediction. The HD model improved the rendering of five organs, including (1) the sternum, (2) the liver, (3) the spleen, (4) the cecum, and (5) the intestine. The white-dashed box highlights a vascular structure. While the 256-sparse image suffers from blurring and discontinuities in the vessel boundaries, the prediction of our HD model recovers sharper, more continuous vascular features, closely resembling the result from 1024-full elements.

**
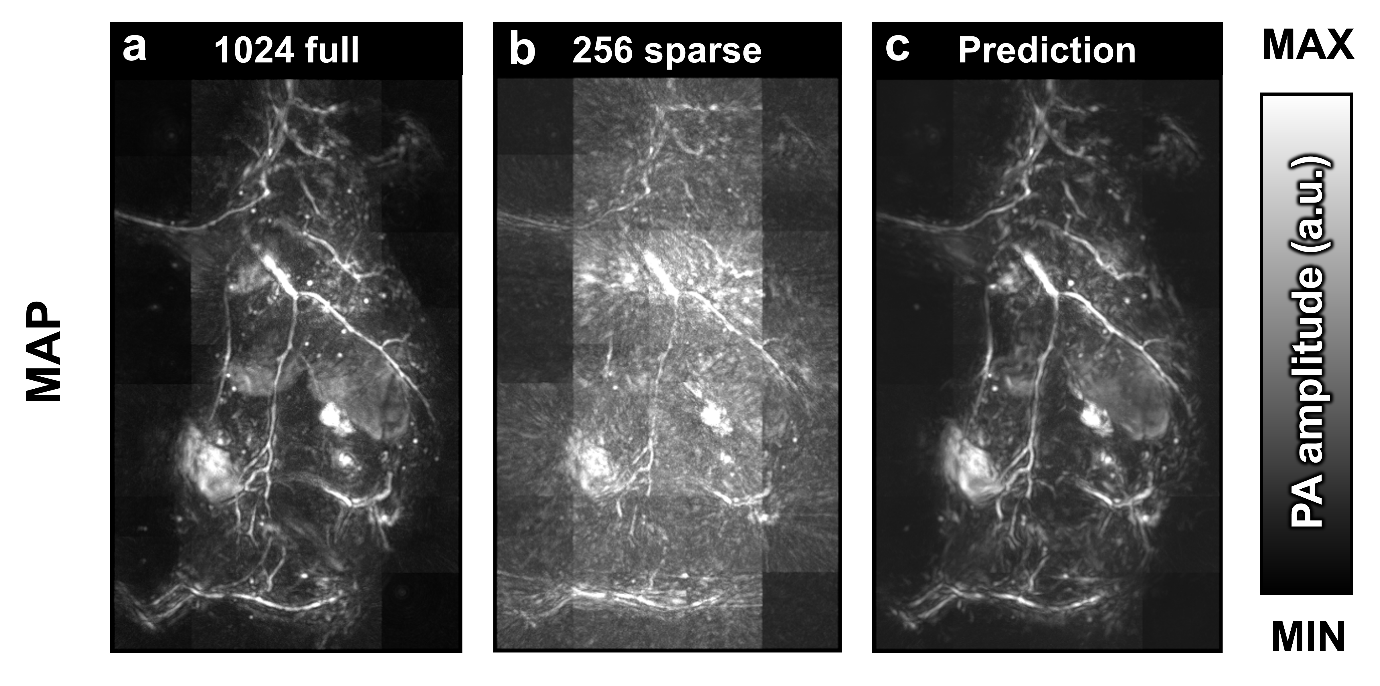
**

**Figure S6. Whole-body mouse MAP images captured in the sagittal plane at 730 nm**. **a)** 1024-full elements. **b)** 256-sparse elements. **c)** HD prediction.


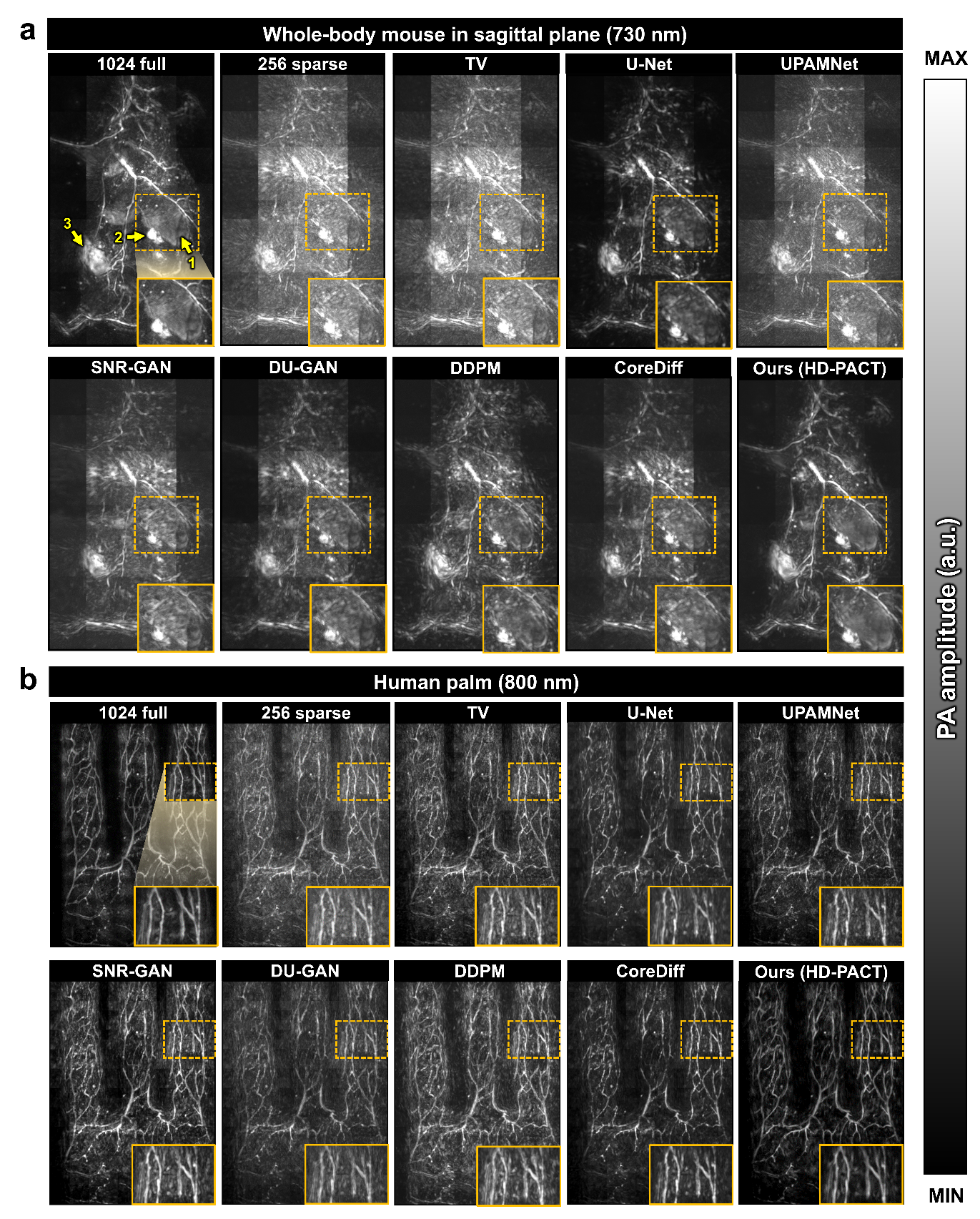


Figure S7. Qualitative comparison of all denoising methods on the sagittal whole-body mouse dataset at 730 nm and the human palm dataset at 800 nm. a) Whole-body sagittal MAP images of a mouse at 730 nm. b) Human palm MAP images at 800 nm. Compared to all other denoising methods, (a) shows the HD-PACT most clearly restores the major organs: the liver (1) (indicated by the yellow-dashed box in Figure S7a, Supporting Information), spleen (2), and intestines (3). (b) shows that, among all denoising methods, HD-PACT most effectively suppresses background noise around the fingers and palm, while accurately recovering fine vascular structures (yellow-dashed box in Figure S7b, Supporting Information) that are comparable in shape and clarity to those in the 1024-full images.


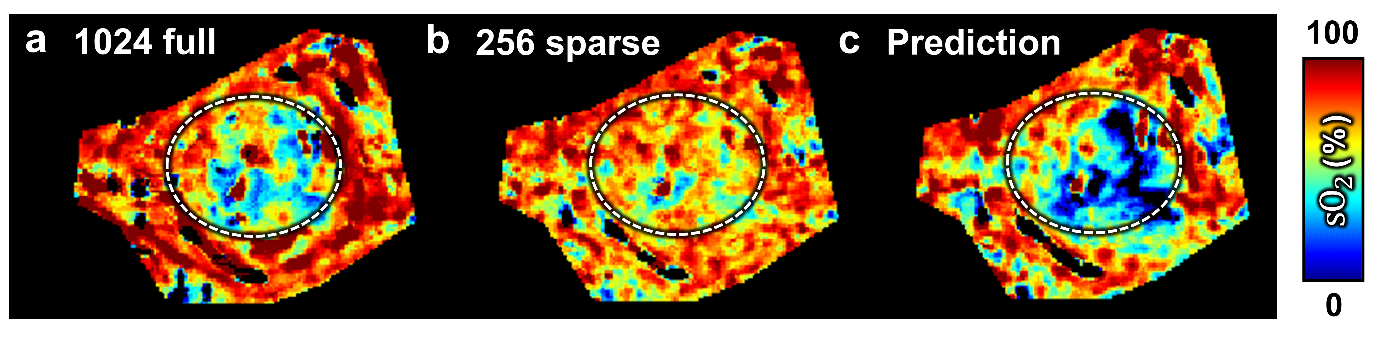


**Figure S8. sO₂ distribution in a B-mode section image of a tumor volume at day 12**. **a)** 1024-full elements. **b)** 256-sparse elements. **c)** HD prediction. The tumor core is marked by the white-dashed circle.


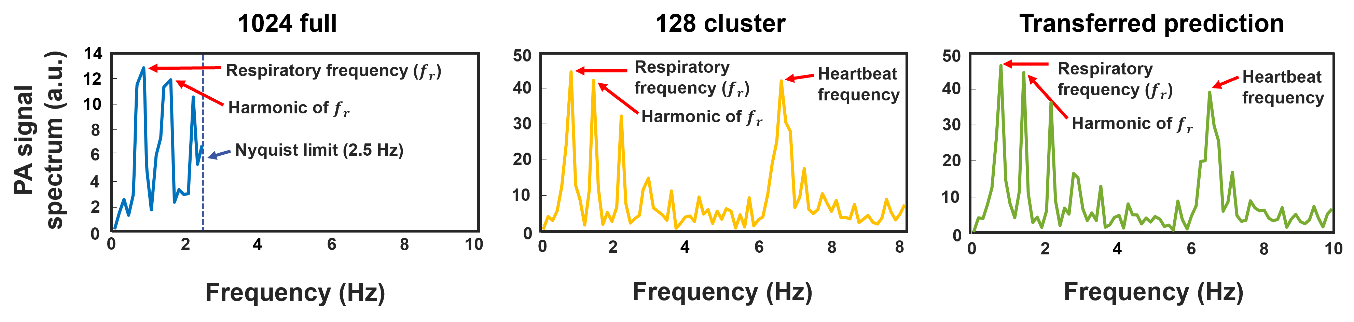
 **Figure S9. Power spectrum of rat heart PA signals using 128-cluster elements.** The power spectrum of time-lapse PA signals in Figure 5c along the frequency axis, highlighting the peak frequencies associated with physiological dynamics.


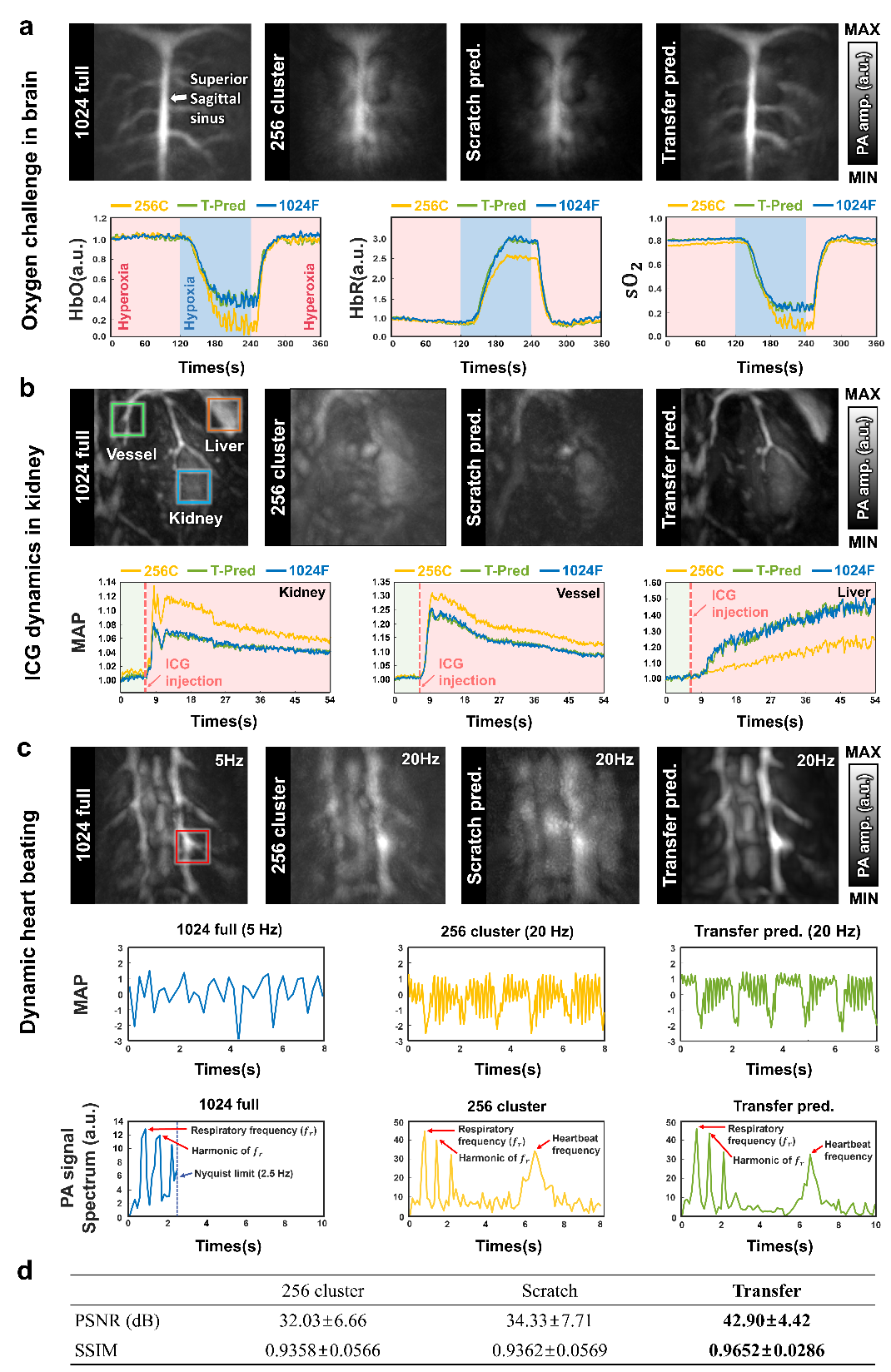


**Figure S10. Dynamic results using transfer learning for a 256-cluster dataset. a)** MAP images of a rat’s brain from 1024-full elements (1024F), 256-cluster elements (256C), the prediction of the scratch-trained HD model, and the prediction of the transfer HD model (T-pred). Time-lapse changes in HbO, HbR, and sO₂ signals during an oxygen challenge are also shown. **b)** MAP images of the kidney (blue box), blood vessel (green box), and liver (orange box), with corresponding time-lapse PA signal changes following ICG injection. **c)** MAP images of a rat’s heart, with time-lapse PA signals and the power spectrum of the heart (red box). Note that due to the limitations of the hardware system, the maximum achievable frame rate for images with 1024-full elements is 5 Hz, while the prediction of the transfer HD from the 256-cluseter data is 20 Hz. **d)** Quantitative comparison of the 256-cluster data, the prediction of the scratch HD model, and the prediction of the transfer HD model to the 1024-full data. The transfer model surpasses both the scratch model and 256-cluster data (p < 0.001).


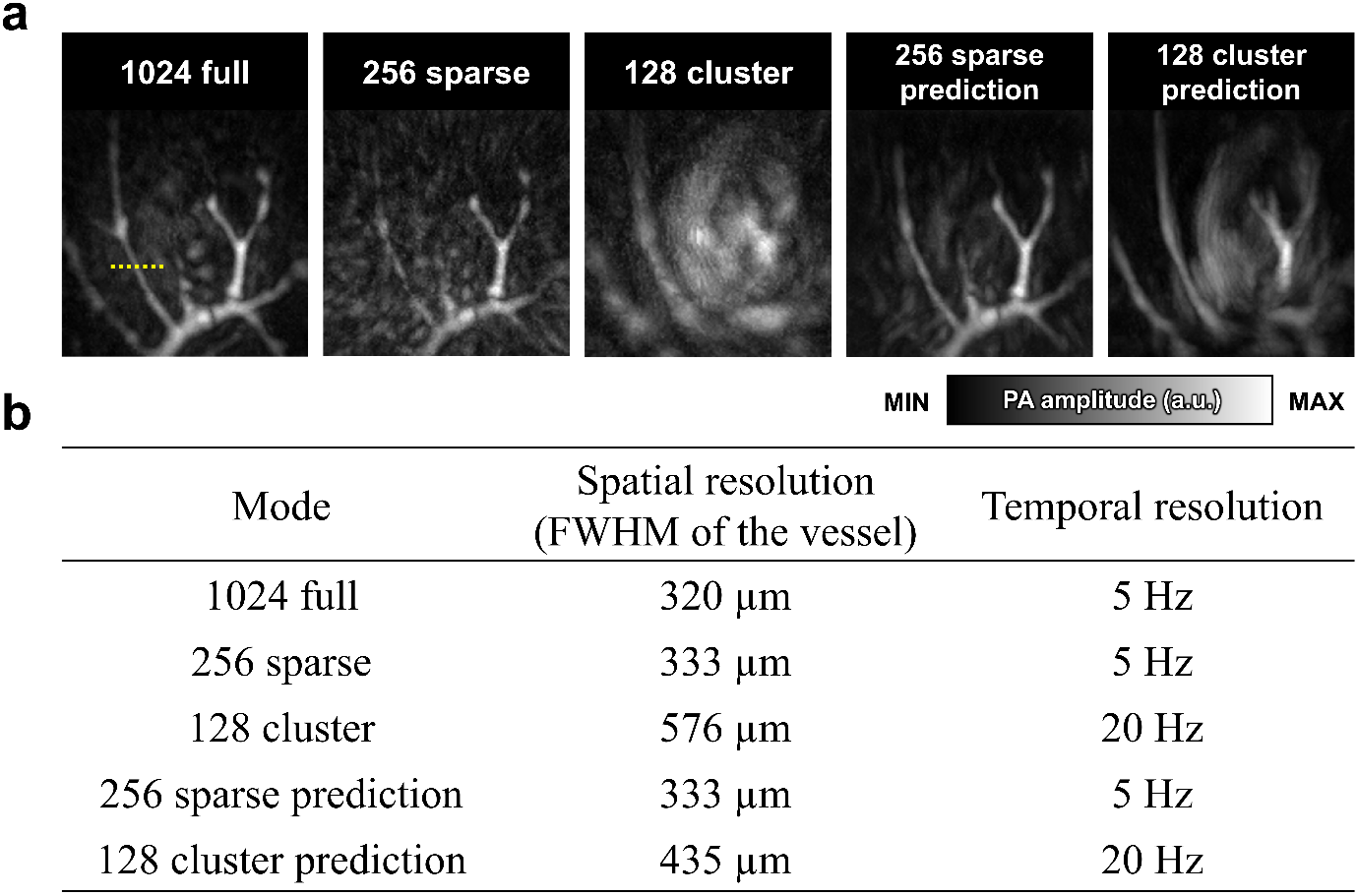


Figure S11. Maximum-amplitude projections of the human palm vessel region and spatiotemporal resolution across imaging modes (full width at half maximum (FWHM)). a) MAP images of vessel ROI patch from the human palm at 900 nm wavelength obtained with 1024-full, 256-sparse, and 128 cluster acquisitions, and their HD predictions (256-sparse prediction, 128-cluster prediction). b) Table summarizing the spatial resolution (FWHM) and temporal resolution for each mode. FWHM was computed from the line-intensity profile taken along the yellow dotted line in the 1024-full image in Figure S11a.


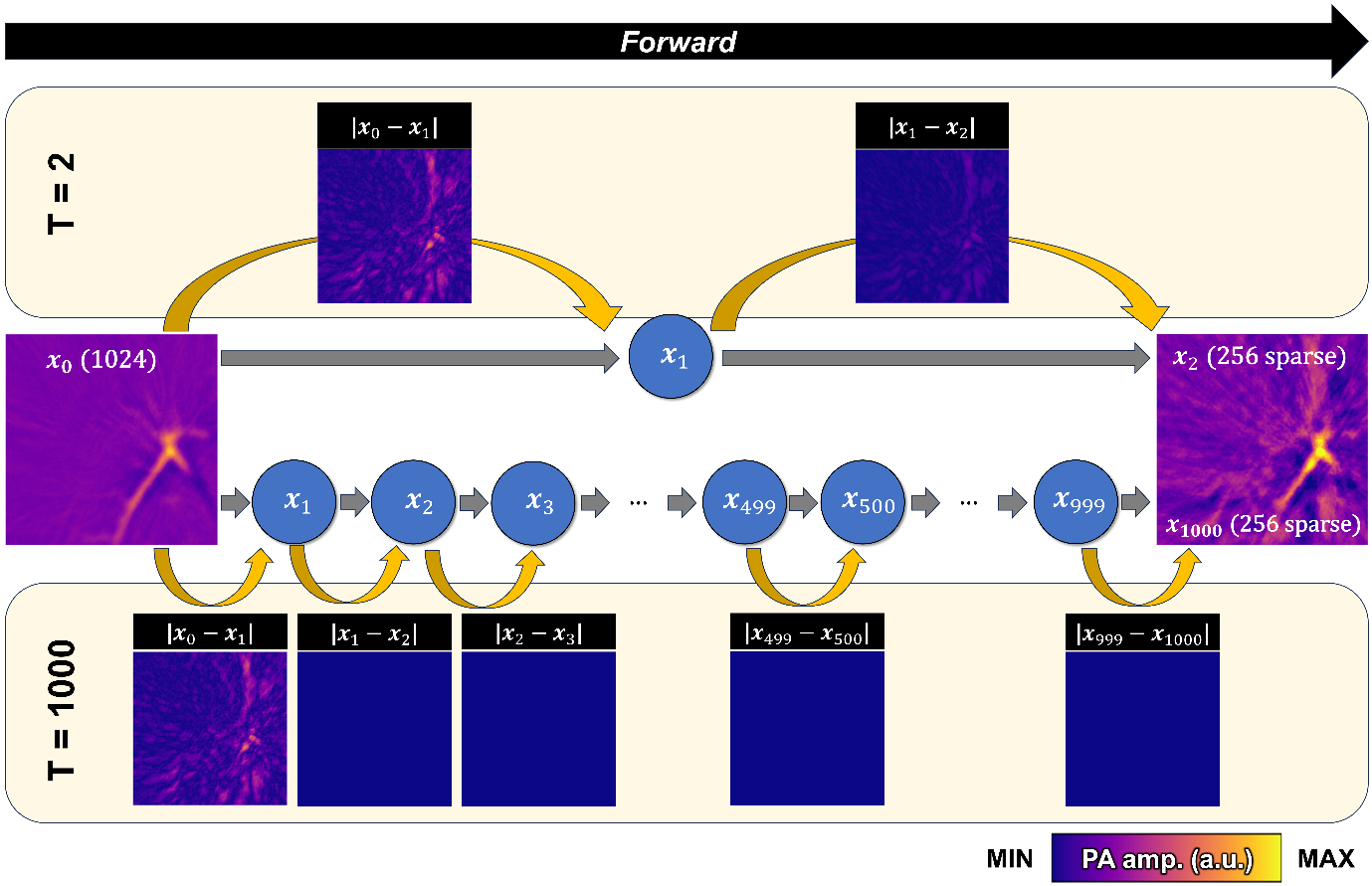


Figure S12. Comparison of difference maps between intermediate states along the forward process ($\boldsymbol{T=2}$ and $\boldsymbol{T=1000}$)


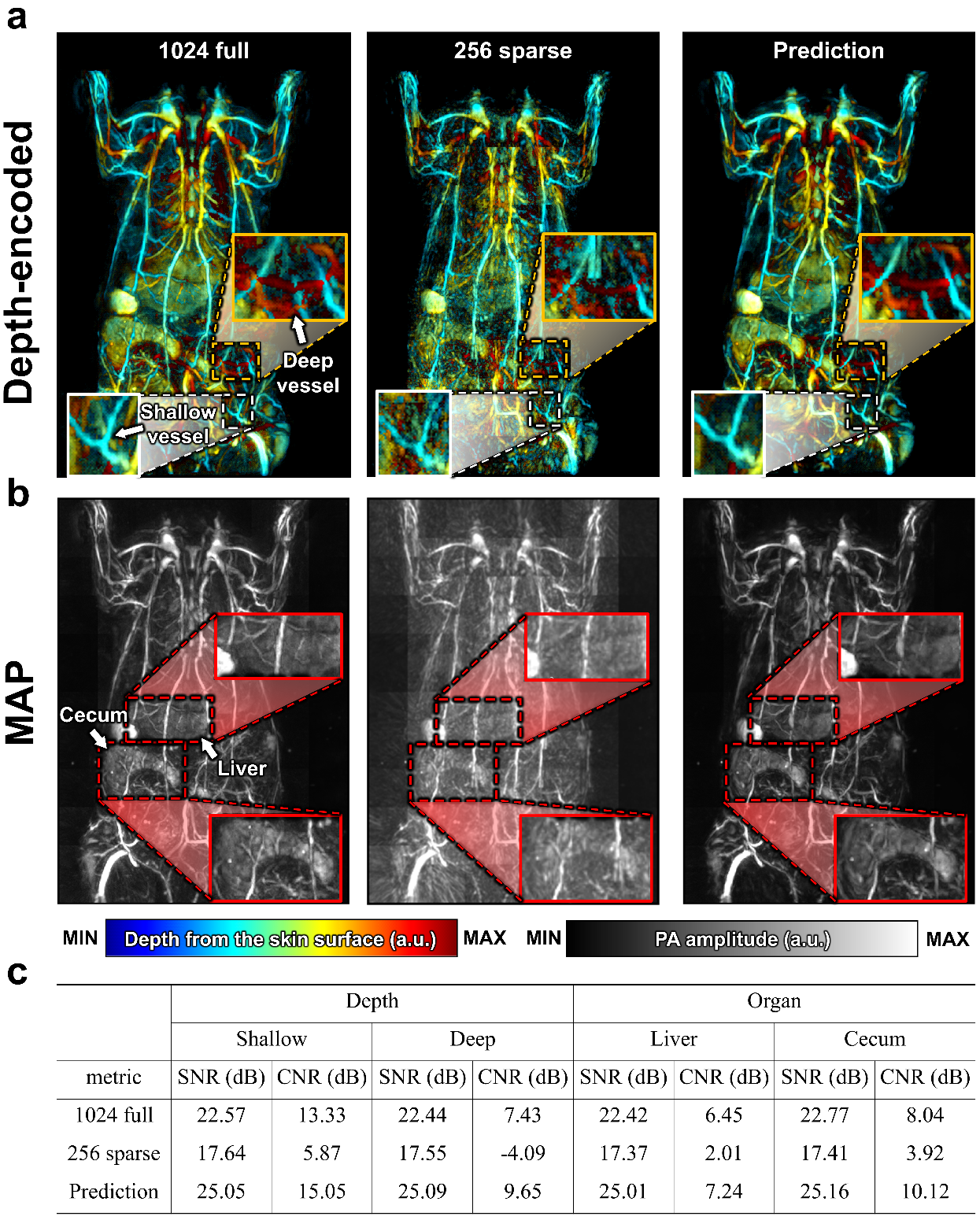


**Figure S13.** **Comparison of SNR and CNR according to depth and organ.** Depth-encoded whole-body rat PACT image at 900 nm was used to select shallow and deep vessels as well as organ ROIs located at similar depths, while SNR and CNR were calculated from the corresponding MAP. **a)** Depth-encoded MAP images obtained with 1024-full, 256-sparse, and the prediction, where the white- and yellow-dashed regions mark the shallow and deep vessel ROIs, respectively. **b)** MAP images corresponding to Figure S13a, with red-dashed boxes indicating organ ROIs (liver and cecum) for quantitative SNR and CNR analysis. **c)** Quantitative comparison of SNR and CNR by depth and organ across 1024-full, 256-sparse, and the prediction. With respect to depth, CNR decreases in deep vessels compared with shallow vessels, with the reduction being most pronounced in the 256-sparse case. With respect to organ, CNR exhibits clear organ-dependent differences. SNR: signal-to-noise ratio, CNR: contrast-to-noise ratio.


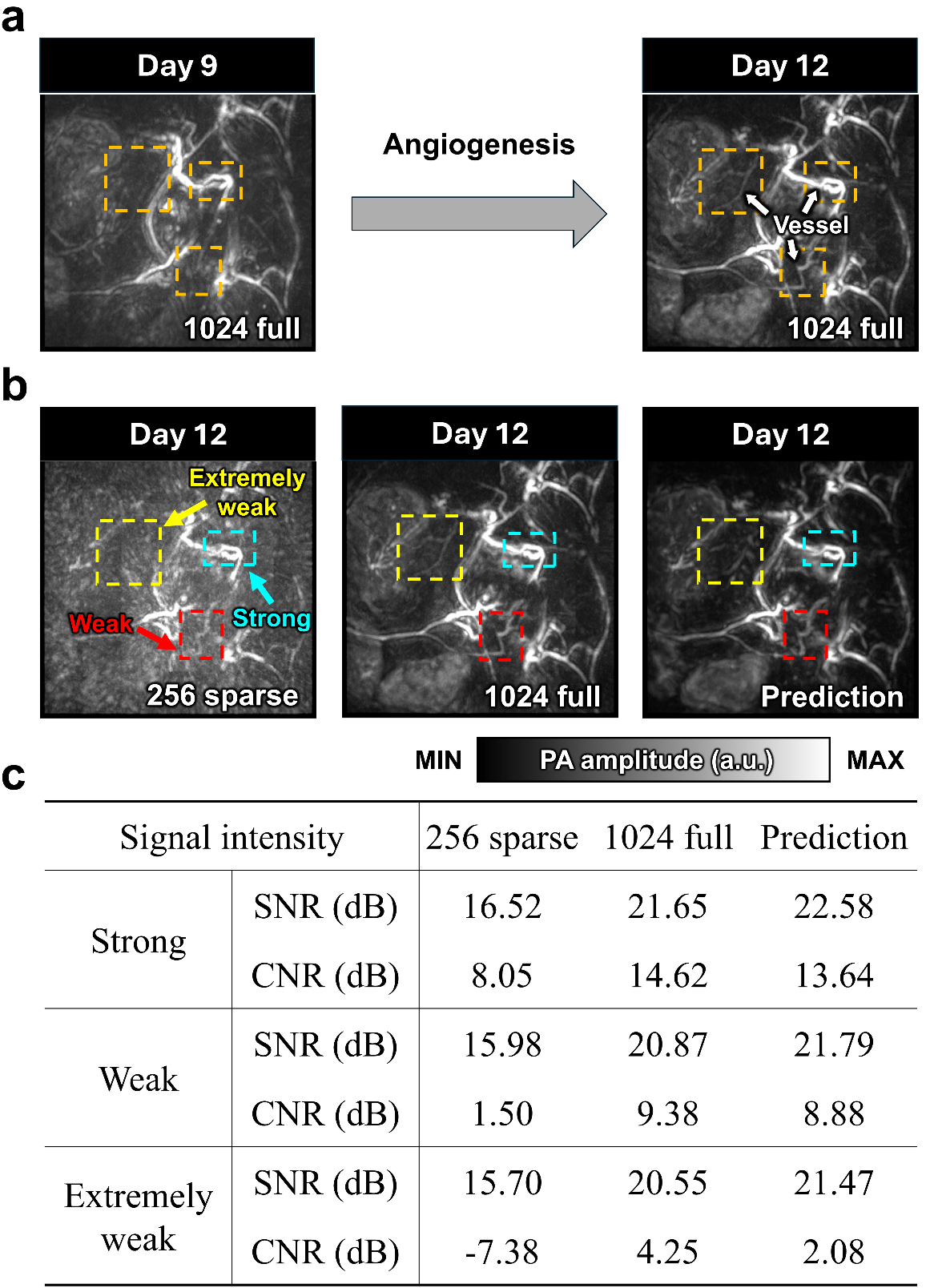


Figure S14. Comparison of SNR and CNR at different signal levels in tumor-induced angiogenesis. a) Tumor ROI MAP images with 1024-fulll elements acquired at 866 nm, corresponding to tumor progression in Figure 4b on day 9 and day 12. In day 12, the yellow dashed boxes delineate newly formed vessel areas associated with tumor-induced angiogenesis. b) Day 12 MAP images across 256-sparse, 1024-full, and prediction acquired at 866 nm. The blue-, red-, and yellow-dashed boxes mark strong, weak, and extremely weak vascular signals, respectively. c) Quantitative SNR and CNR across the three different levels of signal intensity. The vascular signal diminishes from strong to extremely weak, resulting in decreases in both SNR and CNR.

Supplementary Tables

Table S1. Data arrangement for 256-sparse experiments.

| **Train** | | | |
| --- | --- | --- | --- |
| *Type* | *Plane* | *Wavelength (nm)* | *2D slices* |
| Rat1 | Ventral | 900 | 5,760 |
| Rat2 |  |  | 6,912 |
| Rat3 |  |  | 16,128 |
| Rat4 | Dorsal |  | 6,144 |
| Rat5 |  |  | 9,216 |
| Rat6 |  |  | 10,752 |
| **Test** | | | |
| *Type* | *Plane* | *Wavelength (nm)* | *2D slices* |
| Rat7 | Ventral | 900 | 8,960 |
| Rat8 | Dorsal | 900 | 7,680 |
| Tumor day 0 | Sagittal | 730 | 3,584 |
|  |  | 756 |  |
|  |  | 796 |  |
|  |  | 866 |  |
| Tumor day 5 |  | 730 | 2,688 |
|  |  | 756 |  |
|  |  | 796 |  |
|  |  | 866 |  |
| Tumor day 9 |  | 730 | 2,688 |
|  |  | 756 |  |
|  |  | 796 |  |
|  |  | 866 |  |
| Tumor day 12 |  | 730 | 2,688 |
|  |  | 756 |  |
|  |  | 796 |  |
|  |  | 866 |  |
| Human Palm | | 800 | 11,264 |

Table S2. Comparative performances of three key components in the Efficient Hybrid Module on the 256-sparse elements dataset. Compared to the model without an EHM module, the PSNR and SSIM of the model with an EHM (d) are significantly improved (p<0.001) per paired t-test, (a). The best performances are marked in bold.

|  | ECA | SA | ESA | Mamba | PSNR (dB) | SSIM |
| --- | --- | --- | --- | --- | --- | --- |
| (a) | - | - | - | - | 40.71±4.93 | 0.9580±0.0332 |
| (b) | ✓ | ✓ | - | - | 41.37±5.34 | 0.9575±0.0331 |
| (c) | ✓ | - | ✓ | - | 41.70±5.19 | 0.9612±0.0300 |
| (d) | **✓** | ✓ | ✓ | **✓** | **42.20±5.19** | **0.9630±0.0281** |

**Table S3**. **Quantitative comparison of all denoising methods on the entire 256-sparse test dataset.** Across all metrics, our HD-PACT performed the best (p<0.001) in a paired t-test. The best performances are marked in bold.

| **256-sparse elements** | | | |
| --- | --- | --- | --- |
| *Type* | *Method* | *PSNR (dB)* | *SSIM* |
| 256 sparse | | 36.43±5.07 | 0.8875±0.0675 |
| Iterative algorithm-based | TV^2^ | 37.03±5.39 | 0.9025±0.0636 |
| CNN-based | U-Net^3^ | 39.94±4.20 | 0.9582±0.0329 |
|  | UPAMNet^4^ | 38.09±4.65 | 0.9052±0.0589 |
| GAN-based | SNR-GAN^5^ | 37.93±3.93 | 0.9452±0.0402 |
|  | DU-GAN^6^ | 38.57±5.03 | 0.9417±0.0447 |
| Diffusion-based | DDPM-1000^7^ | 36.06±7.16 | 0.9539±0.0349 |
|  | CoreDiff-10^1^ | 40.36±5.54 | 0.9491±0.0413 |
|  | **HD-PACT (Ours)** | **42.20±5.19** | **0.9630±0.0281** |

Table S4. Quantitative comparison of all denoising methods on the sagittal whole-body mouse dataset at 730 nm and the human palm dataset at 800 nm. Across all metrics, our HD-PACT performed the best in both datasets (p<0.001, paired t-test). The best performances are marked in bold.

| **Sagittal whole-body mouse dataset (730 nm)** | | | |
| --- | --- | --- | --- |
| *Type* | *Method* | *PSNR (dB)* | *SSIM* |
| 256 sparse | | 37.82±5.06 | 0.9030±0.0666 |
| Iterative algorithm-based | TV^2^ | 38.56±5.47 | 0.9168±0.0626 |
| CNN-based | U-Net^3^ | 41.80±4.27 | 0.9631±0.0312 |
|  | UPAMNet^4^ | 39.23±4.83 | 0.9160±0.0588 |
| GAN-based | SNR-GAN^5^ | 40.12±4.90 | 0.9388±0.0462 |
|  | DU-GAN^6^ | 39.70±3.33 | 0.9509±0.0403 |
| Diffusion-based | DDPM-1000^7^ | 41.98±5.11 | 0.9593±0.0351 |
|  | CoreDiff-10^1^ | 42.61±5.31 | 0.9618±0.0328 |
|  | **HD-PACT (Ours)** | **43.53±5.28** | **0.9661±0.0285** |
| **Human palm (800 nm)** | | | |
| 256 sparse | | 33.65±3.90 | 0.8578±0.0657 |
| Iterative algorithm-based | TV^2^ | 34.04±4.11 | 0.8729±0.0635 |
| CNN-based | U-Net^3^ | 35.19±2.81 | 0. 9318±0.0418 |
|  | UPAMNet^4^ | 35.73±3.79 | 0. 8835±0.0569 |
| GAN-based | SNR-GAN^5^ | 26.15±0.95 | 0. 7118±0.0693 |
|  | DU-GAN^6^ | 31.59±1.63 | 0. 8950±0.0554 |
| Diffusion-based | DDPM-1000^7^ | 26.28±0.42 | 0. 9311±0.0414 |
|  | CoreDiff-10^1^ | 34.64±3.60 | 0. 9019±0.0493 |
|  | **HD-PACT (Ours)** | **38.29±3.88** | **0. 9429±0.0339** |

Table S5. Quantitative comparison of the transfer HD model with the scratch HD model across individual 128-cluster elements datasets. Across all datasets, the transfer HD model performed the best (p < 0.001, paired t-test) in terms of both PSNR and SSIM. The best performances are marked in bold.

| *Dataset* | *Method* | *PSNR (dB)* | *SSIM* |
| --- | --- | --- | --- |
| Brain | 128 cluster | 29.20±2.10 | 0.9481±0.0433 |
|  | Scratch | 37.91±5.61 | 0.9552±0.0379 |
|  | **Transfer** | **43.55±3.59** | **0.9739±0.0171** |
| Organs  (Kidney, vessel,  and liver) | 128 cluster | 25.34±1.76 | 0.8523±0.0707 |
|  | Scratch | 29.40±2.49 | 0.8575±0.0659 |
|  | **Transfer** | **38.27±2.46** | **0.9251±0.0256** |
| Heart  (5 Hz) | 128 cluster | 24.29±1.04 | 0.9222±0.0546 |
|  | Scratch | 34.70±5.95 | 0.9332±0.0489 |
|  | **Transfer** | **38.79±4.03** | **0.9490±0.0324** |

**References**

1. Gao, Q., Li, Z., Zhang, J., Zhang, Y. & Shan, H. CoreDiff: Contextual error-modulated generalized diffusion model for low-dose CT denoising and generalization. *IEEE Transactions on Medical Imaging* (2023).

2. Boigné, E., Parkinson, D.Y. & Ihme, M. Towards data-informed motion artifact reduction in quantitative CT using piecewise linear interpolation. *IEEE Transactions on Computational Imaging* **8**, 917-932 (2022).

3. Ronneberger, O., Fischer, P. & Brox, T. U-net: Convolutional networks for biomedical image segmentation. in *Medical image computing and computer-assisted intervention–MICCAI 2015: 18th international conference, Munich, Germany, October 5-9, 2015, proceedings, part III 18* 234-241 (Springer, 2015).

4. Liu, Y.*, et al.* UPAMNet: A unified network with deep knowledge priors for photoacoustic microscopy. *Photoacoustics* **38**, 100608 (2024).

5. Momen-Tayefeh, M., Momen-Tayefeh, M., Hasheminasab, F.Z. & Ghahramani, S.A.G. SNRGAN: The Semi Noise Reduction GAN for Image Denoising. in *2024 20th CSI International Symposium on Artificial Intelligence and Signal Processing (AISP)* 1-5 (IEEE, 2024).

6. Huang, Z., Zhang, J., Zhang, Y. & Shan, H. DU-GAN: Generative adversarial networks with dual-domain U-Net-based discriminators for low-dose CT denoising. *IEEE Transactions on Instrumentation and Measurement* **71**, 1-12 (2021).

7. Ho, J., Jain, A. & Abbeel, P. Denoising diffusion probabilistic models. *Advances in neural information processing systems* **33**, 6840-6851 (2020).
